# Neutralization-guided design of HIV-1 envelope trimers with high affinity for the unmutated common ancestor of CH235 lineage CD4bs broadly neutralizing antibodies

**DOI:** 10.1371/journal.ppat.1008026

**Published:** 2019-09-17

**Authors:** Celia C. LaBranche, Rory Henderson, Allen Hsu, Shay Behrens, Xuejun Chen, Tongqing Zhou, Kevin Wiehe, Kevin O. Saunders, S. Munir Alam, Mattia Bonsignori, Mario J. Borgnia, Quentin J. Sattentau, Amanda Eaton, Kelli Greene, Hongmei Gao, Hua-Xin Liao, Wilton B. Williams, James Peacock, Haili Tang, Lautaro G. Perez, Robert J. Edwards, Thomas B. Kepler, Bette T. Korber, Peter D. Kwong, John R. Mascola, Priyamvada Acharya, Barton F. Haynes, David C. Montefiori

**Affiliations:** 1 Department of Surgery, Duke University Medical Center, Durham, NC, United States of America; 2 Duke Human Vaccine Institute, Duke University Medical Center, Durham, NC, United States of America; 3 Genome Integrity and Structural Biology Laboratory, National Institute of Environmental Health Sciences, National Institutes of Health, Department of Health and Human Services, Research Triangle Park, NC, United States of America; 4 Vaccine Research Center, National Institute of Allergy and Infectious Diseases, National Institutes of Health, Bethesda, Maryland, United States of America; 5 Department of Medicine, Duke University Medical Center, Durham, NC, United States of America; 6 The Sir William Dunn School of Pathology, University of Oxford, Oxford, United Kingdom; 7 Department of Microbiology, Boston University School of Medicine, Boston, MA, United States of America; 8 Los Alamos National Laboratory, Theoretical Biology & Biophysics, Los Alamos, NM, United States of America; University of Wisconsin, UNITED STATES

## Abstract

The CD4 binding site (CD4bs) of the HIV-1 envelope glycoprotein is susceptible to multiple lineages of broadly neutralizing antibodies (bnAbs) that are attractive to elicit with vaccines. The CH235 lineage (VH1-46) of CD4bs bnAbs is particularly attractive because the most mature members neutralize 90% of circulating strains, do not possess long HCDR3 regions, and do not contain insertions and deletions that may be difficult to induce. We used virus neutralization to measure the interaction of CH235 unmutated common ancestor (CH235 UCA) with functional Env trimers on infectious virions to guide immunogen design for this bnAb lineage. Two Env mutations were identified, one in loop D (N279K) and another in V5 (G458Y), that acted synergistically to render autologous CH505 transmitted/founder virus susceptible to neutralization by CH235 UCA. Man_5_-enriched *N*-glycans provided additional synergy for neutralization. CH235 UCA bound with nanomolar affinity to corresponding soluble native-like Env trimers as candidate immunogens. A cryo-EM structure of CH235 UCA bound to Man_5_-enriched CH505.N279K.G458Y.SOSIP.664 revealed interactions of the antibody light chain complementarity determining region 3 (CDR L3) with the engineered Env loops D and V5. These results demonstrate that virus neutralization can directly inform vaccine design and suggest a germline targeting and reverse engineering strategy to initiate and mature the CH235 bnAb lineage.

## Author summary

Despite a wealth of information on the epitopes, ontogeny, structure and maturation pathways of multiple epitope classes of HIV-1 broadly neutralizing antibodies (bnAbs), there has been little progress eliciting similar antibodies by vaccination. One major contributing factor is the failure of many candidate immunogens to engage germline reverted forms of bnAbs, making it unlikely that they will provide adequate stimulation of appropriate naïve B cells to initiate bnAb lineages. Here we used virus neutralization to identify two point mutations and a modified glycan profile that together render HIV-1 CH505 Env-pseudotyped virus highly susceptible to neutralization by a germline-reverted form of the CH235 lineage of CD4 binding site (CD4bs) bnAbs. These same modifications permit strong binding of corresponding soluble native-like CH505 Env trimers to germline-reverted CH235. These observations provide a conceptual framework for the design and testing of novel immunogens that aim to elicit the CH235 bnAb lineage.

## Introduction

The trimeric HIV-1 envelope glycoprotein (Env) spikes that mediate virus entry into cells are vulnerable to at least six distinct epitope classes of broadly neutralizing antibodies (bnAbs), where each bnAb class is a major focus for vaccine design [1]. These bnAbs arise during chronic HIV-1 infection and target epitopes in the CD4 binding site (CD4bs), V2-apex and V3-glycan regions of gp120, the membrane-proximal external region (MPER) of gp41, the gp120-gp41 interface/gp41 fusion peptide, and glycan-dependent epitopes in the center of the gp120 “silent face” [2]. Efforts to elicit HIV-1 bnAbs by vaccination face unusual challenges, due in part to the glycan shielding and conformational masking mechanisms the virus uses to evade antibody recognition [3, 4]. Other challenges include proper engagement of the correct precursor B cells that give rise to bnAbs [5, 6], overcoming host tolerance controls of bnAb development [7, 8, 9], and an ability to drive high levels of somatic hypermutation that are often required for bnAb activity [10, 11]. While there has been modest success inducing bnAb-like activity in animal models [12, 13, 14, 15], much stronger and broader neutralization will be needed to provide adequate protection in humans.

New information is constantly emerging on the structures of bnAbs and their epitopes, as well as maturation pathways that give rise to bnAbs in HIV-1-infected people [16]. This information is useful for engineering novel immunogens with a focus on inducing either specific bnAb lineages, or general epitope classes of bnAbs [17]. The design and selection of candidate immunogens is often guided in part by measures of binding to one or more germline-reverted, intermediate or mature forms of bnAbs as indicators of epitope integrity [18, 19, 20]. Much attention focuses on immunogens that preserve and stabilize native Env trimer structure to favor correct antibody maturation while minimizing off-target antibody responses [21]. Considerable progress has been made in designing and producing stabilized, soluble native-like trimers as candidate immunogens [22, 23, 24, 25, 26, 27]. Unlike these stabilized trimers, unliganded native trimers on the virus surface are structurally dynamic, spontaneously transitioning through at least three conformational states [28]. It is not yet clear how these dynamic properties will impact the conformation(s) needed for desired immunogenicity.

We recently used Env-pseudotyped virus neutralization to guide the design of germline-targeting immunogens for VRC01-class bnAbs [29]. Neutralization by germline-reverted VRC01 (VRC01gl) was used to predict precursor B cell receptor engagement by functional Env trimers on HIV-1 strain 426c. Neutralization by VRC01gl required targeted deletion of one or more *N*-glycans bordering the CD4bs, combined with Man_5_-enrichment of remaining *N*-glycans that are otherwise processed into larger complex-type glycans. These changes aimed to facilitate VRC01gl binding by increasing exposure of the CD4bs while having minimal impact on overall native Env trimer structure. While this approach worked for germline-reverted forms of several VRC01-class bnAbs, it was not successful for germline-reverted forms of other CD4bs bnAbs.

Here we have explored a similar approach by using autologous virus neutralization to guide the design of immunogens aiming to elicit the CH103 and CH235 lineages of CD4bs bnAbs. These two lineages co-evolved in a clade C HIV-1 infected individual (CH505) in Africa who was longitudinally sampled from acute infection for a period of 6 years [30]. CH103 utilizes VH4-59 and matured to neutralize 55% of a multi-clade panel of 196 Env-pseudotyped viruses [30]. CH235 and its more mature lineage member, CH235.12, utilize VH1-46, neutralize 18% and 90%, respectively, of a multi-clade panel of 199 Env-pseudotyped viruses, and do not require insertions or deletions for this neutralizing activity [31, 32]. The inferred unmutated common ancestor (UCA) of the CH235/CH235.12 lineage, referred to here as CH235 UCA2, exhibits micromolar affinity binding and weak neutralizing activity against an early autologous CH505 Env that differs from the transmitted-founder (TF) Env by a single N279K change, suggesting that this variant (also called M5) initiated the lineage [31].

We describe a discrete set of synergistic Env modification that render autologous CH505TF Env-pseudotyped virus highly susceptible to neutralization by CH235 UCA2 in a stepwise manner. Moreover, neutralization potency against engineered Env-pseudotyped viruses correlated with binding affinity to corresponding soluble native-like Env trimers as candidate immunogens.

## Results

### Targeted glycan deletion and Man_5_-enrichment

**Fig 1:**
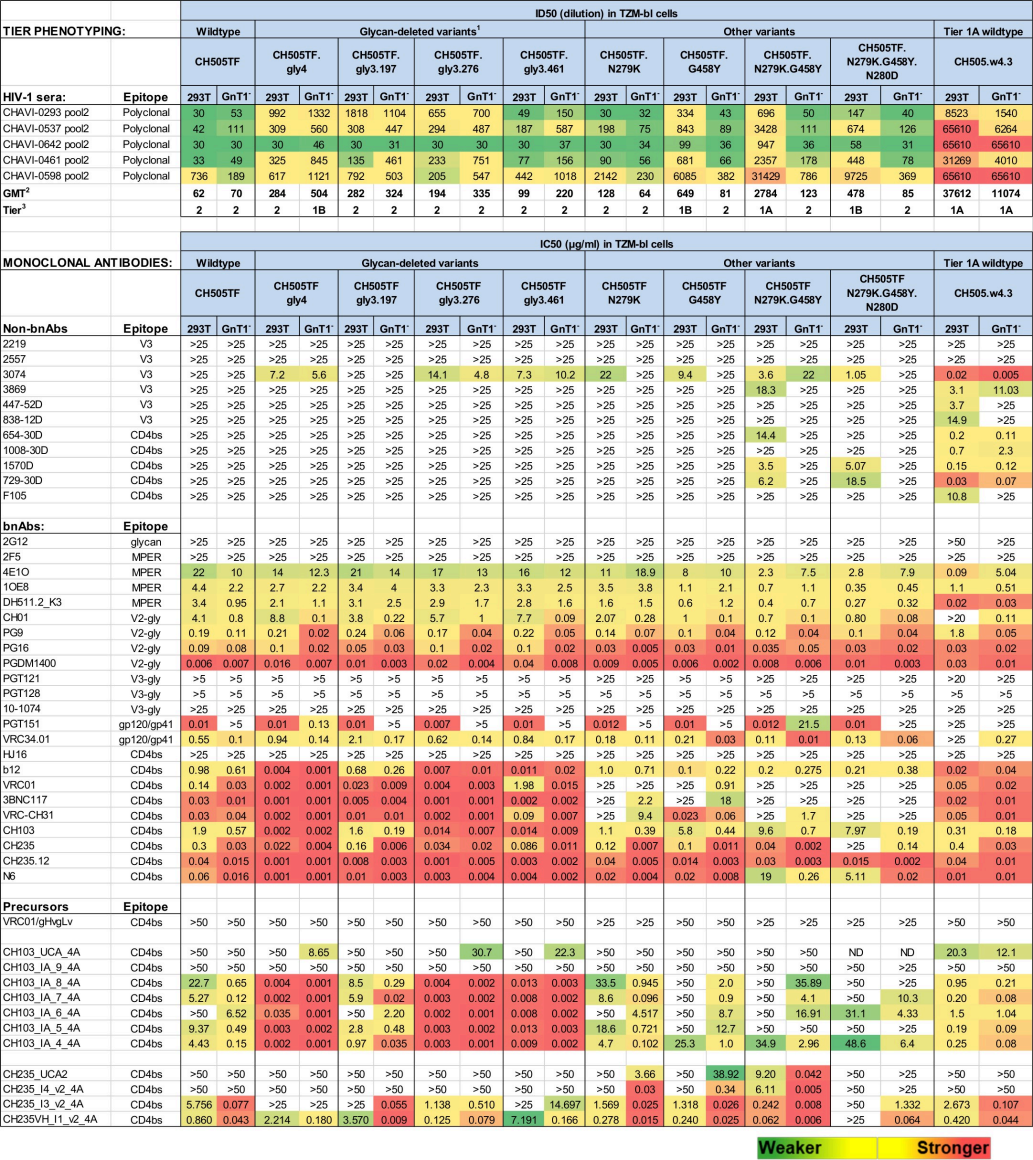
Neutralization phenotype of parental and mutants of CH505TF produced in 293T and 293S GnT1^-^ cells. Neutralization tier phenotypes were characterized with pooled serum samples from 5 HIV-1 infected individuals (CHAVI samples). Additional neutralization phenotyping was performed with monoclonal antibodies. ^1^CH505TF.gly4 (197, 461/462, 276, 362); CH505TF.gly3.197 (461/462, 276, 362); CH505TF.gly3.276 (197, 461/462, 362); CH505TF.gly3.461 (197, 276, 362). ^2^GMT, geometric mean titer. ^3^Tier classification: Tier 1A (ID50 >2000), Tier 1B (ID50 350–2000), Tier 2 (ID50 50–349), Tier 3 (ID50 <50). ND, not done. Neutralization curves for the UCAs and intermediates of CH103 and CH235 are shown in S1A–S1K Fig.

We sought to identify Env modifications that would render CH505TF Env susceptible to neutralization by the UCAs and intermediates of the CD4bs bnAbs CH103 and CH235. We began by testing autologous CH505TF Env-pseudotyped viruses that lacked select glycans bordering the CD4bs. One variant lacked four glycans at N197, N461/462, N276 and N362 (gly4), whereas the others lacked three glycans by adding back either N197 (gly3.197), N276 (gly3.276) or N461 (gly3.461) [33]. As reported previously [33], glycan-deleted CH505TF was highly sensitive to neutralization by mature CD4bs bnAbs, while glycan deletion had little impact on most other mature bnAbs (Fig 1).

A notable exception was that all Man_5_-enriched viruses exhibited increased resistance to PGT151, which agrees with the known dependency of this interface bnAb on complex-type glycans [34, 35]. Also as reported previously [33], the gly4, gly3.276 and gly3.461 variants were 136–950 times more sensitive to mature CH103, and 3.5–40 times more sensitive to mature CH235 and CH235.12 than parental CH505TF (Fig 1). In addition, we found that gly4, gly3.276 and gly3.461 were substantially more sensitive to intermediates of CH103 but not intermediates of CH235 (Fig 1). Notably, glycan deletion alone was not sufficient for neutralization by the UCAs of either bnAb lineage (Fig 1).

Our previous work showed that neutralization of 426c by germline-reverted VRC01 required both targeted glycan deletion and Man_5_-enrichment [29]; therefore, we sought to determine whether Man_5_-enrichment of glycan-deleted CH505TF would enable neutralization by the UCAs of CH103 and CH235/CH235.12. Parental and glycan-deleted CH505TF Env-pseudoviruses were produced in 293S/GnT1^-^ cells to enrich for Man_5_GlcNAc_2_ (Man_5_) glycoforms of *N*-linked glycans that are otherwise processed into larger complex-type glycans. These cells lack the enzyme *N-*acetylglucosaminyltransferase 1 (GnT1^-^) that is responsible for attachment of GlcNAc to Man_5_GlcNAc_2_ in the medial-Golgi as a requisite step for complete processing [36]. Man_5_-enrichment has potential to reduce steric barriers to germline bnAb binding without disrupting native Env conformation [29, 37, 38]. GnT1^-^ versions of the CH505TF variants tested here were often less infectious than their 293T counterparts but nonetheless remained adequately infectious for neutralization assays in TZM-bl cells (S1 Table), where only fusion-competent Env trimers are targets for neutralization [39]. Moreover, GnT1^-^ production of the glycan-deleted viruses resulted in only modest increases in sensitivity to neutralization by HIV-1 sera, and did not render the viruses sensitive to most non-bnAbs tested (Fig 1), indicating that Man_5_-enrichment did not expose cryptic epitopes that are primary targets for neutralization on tier 1A viruses [40].

When assayed in TZM-bl cells, Man_5_-enriched CH505TF.gly4, CH505TF.gly3.276 and CH505TF.gly3.461 were moderately sensitive to neutralization by CH103 UCA (Fig 1, S1A Fig). In addition, Man_5_-enrichment of CH505TF and CH505TF.gly3.197 resulted in >10-fold increased neutralization by intermediates 4–8 of CH103 (Fig 1, S1C–S1G Fig). Despite these advantages for the UCA and intermediates of CH103, Man_5_-enrichment did not permit neutralization by CH235 UCA2 or the earliest CH235 intermediate tested (CH235_I4).

### N279K and G458Y mutations

**Fig 2:**
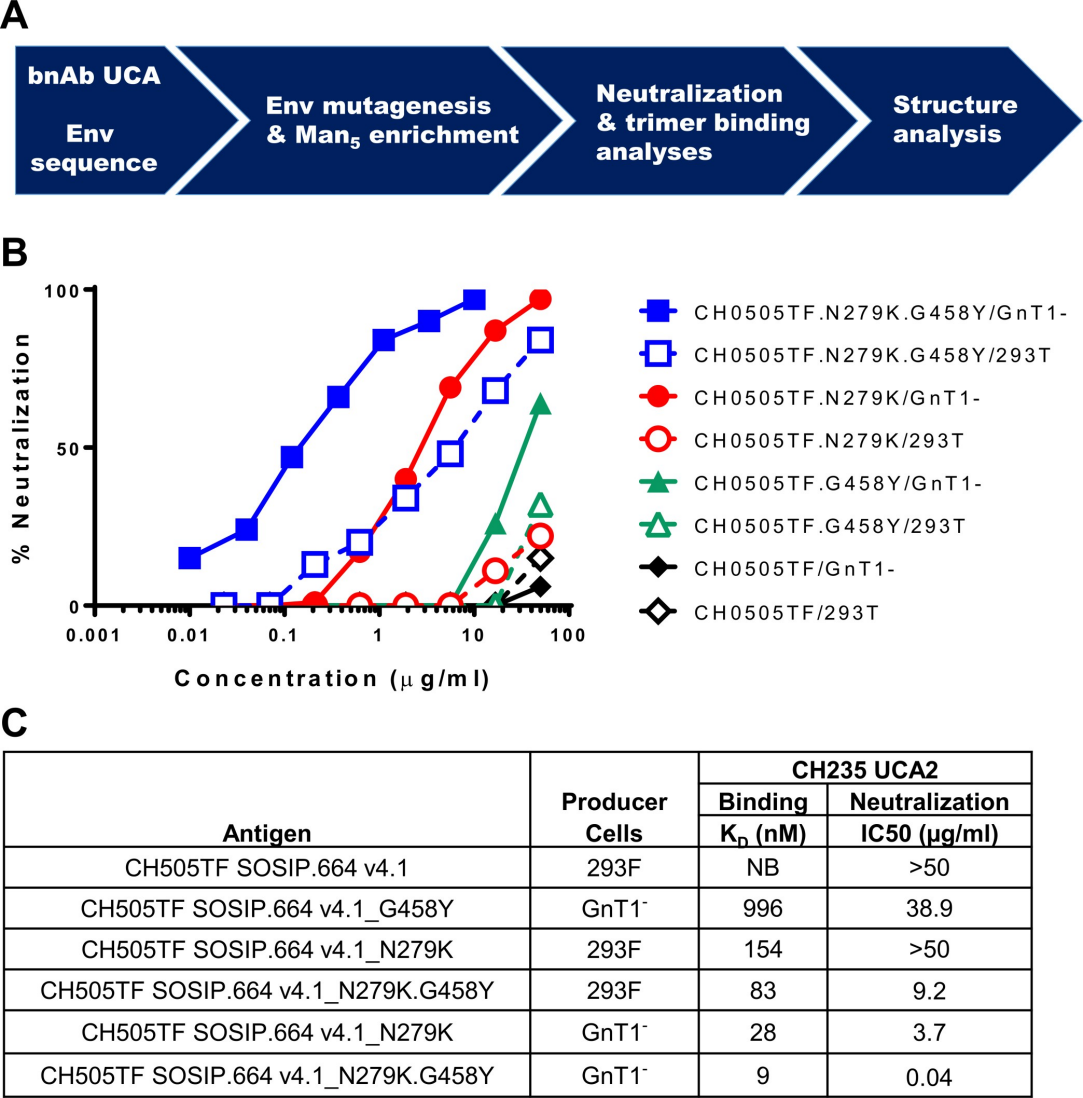
Neutralization by CH235 UCA2 predicts graded binding to corresponding SOSIP trimers. (A) Overall strategy to identify and characterize engineered CH505TF Envs that engage CH235 UCA2. (B) Neutralization of parental and mutant CH505TF Env-pseudotyped viruses produced in either 293T or 293S GnTI^-^ cells and assayed with CH235 UCA2 in TZM-bl cells. Shown are representative curves from S1H Fig. (C) SPR binding of CH235 UCA2 to parental and mutant CH505TF SOSIP trimers produced in either Freestyle293 (293F) or 293S GnT1^-^ cells. The apparent dissociation rate constant (K_D_) values are an average of two measurements (see S3 Fig). NB, no binding.

In an attempt to overcome the barrier to CH235 UCA2 neutralization, we combined Man_5_-enrichment with an N279K change in gp120 loop D that triggered the CH235 lineage in donor CH505. The N279K has been previously reported as the natural CH505 M5 variant of the CH505 transmitted/founder virus [32]. Another mutation, G458Y (V5 proximal), was found to make CH505TF/GnT1^-^ moderately sensitive to neutralization by CH235 UCA2. Both mutations are resistance mutations for VRC01-class bnAbs [41, 42, 43, 44] but neither one imparts resistance to CH103, CH235 and CH235.12 (Fig 1). We examined N279K and G458Y in the context of CH505TF rather than the glycan deleted variants because glycan deletion provided little or no benefit and sometimes was detrimental for neutralization by CH235 intermediates I3 and I1 (Fig 1). The double mutation (N279K.G458Y) in CH505TF was synergistic in overcoming the barrier to CH235 UCA2 neutralization, with additional synergy provided by Man_5_-enrichment, resulting in a remarkable IC50 of 0.04 μg/ml against the Man_5_-enriched double mutant (Fig 1, Fig 2B and 2C, S1H Fig).

Neutralization potency was reduced 230-fold when the double mutant was not Man_5_-enriched (IC50 = 9.2 μg/ml), illustrating a strong synergistic effect of Man_5_ glycans. As single mutants, Man_5_-enriched N279K and G458Y were 92-fold and 973-fold less susceptible, respectively, than the Man_5_-enriched double mutant, illustrating potent synergy between the two mutations. Neither single mutant was neutralized >50% when not Man_5_-enriched. These combined observations point to a set of Env modifications that predict different magnitudes of interaction with CH235 UCA2. Several observations indicate that these interactions are not dependent on an open trimer conformation. Thus, Man_5_-enriched mutant viruses exhibited a tier 2 neutralization phenotype that is typical of a closed Env conformation yet is neutralized by CH235 UCA2. In addition, CH235 UCA2 did not neutralize the tier 1A virus CH505.w4.3, which represents an open conformation (Fig 1) [40]. Finally, the double mutant produced in 293T cells, which exhibits a tier 1A phenotype, was far less susceptible to CH235 UCA2 neutralization than the GnT1- version of this virus, which exhibits a tier 2 phenotype (Fig 1).

These two mutations, N279K and G458Y, also had an impact on intermediates of CH235. Man_5_-enriched N279K, G458Y and N279K.G458Y mutants were approximately 100–10,000-fold more sensitive to I4, and were 1.7–9.6-fold more sensitive to later intermediates of CH235 compared to Man_5_-enriched CH505TF (Fig 1, S1I–S1K Fig). These mutations had little or no impact on neutralization by VRC01gl, CH103 UCA and intermediates of CH103 compared to corresponding CH505TF produced in 293T and GnT1^-^ cells (Fig 1, S1A–S1G Fig).

The remarkable neutralization of the N279K.G458Y virus by CH235 UCA2 led us to investigate whether further optimization was possible by substituting other amino acids at these two positions. Some amino acids were lethal to the virus and could not be tested. Among the substitutions at position 279 that were testable as Man_5_-enriched viruses, none proved superior to N279K (S2 Table). On the other hand, two substitutions at position 458 (G458C and G458L) provided modest improvement over G458Y as single mutants (S2 Table). Nonetheless, combining either G458C or G458L with N279K provided only 2-3-fold improved susceptibility to CH235 UCA2 and no improved susceptibility to the CH235 intermediates compared to N279K.G458Y (S2 Table).

### CH235 UCA2 neutralization potency correlates with SOSIP binding

Because Env-pseudotyped viruses are not suitable as vaccine immunogens, we tested whether our findings translate to recombinant soluble native-like SOSIP.664 gp140 trimers as an alternate vaccine platform. The CH505TF SOSIP gp140 was stabilized by creating a chimeric SOSIP that contained select BG505 residues in gp120 and gp41 [33, 45]. Additionally, E64K and A316W were introduced to stabilize the Env in the closed conformation [23, 45]. Stabilized SOSIP versions of mutated and parental CH505TF were produced in Freestyle293 (293F) and 293S/GnT1^-^ cells (S2 Fig), and relative binding affinities to CH235 UCA2 were measured by Surface Plasmon Resonance (SPR) (S3 Fig). CH235 UCA2 bound with high affinity (9 nM) to Man_5_-enriched CH505.N279K.G458Y, and bound with incrementally lower affinities to the other antigens, as predicted by neutralization potency against corresponding Env-pseudotyped viruses (Fig 2C, S3 Fig). These results suggest that stabilized SOSIP.664 trimers may be a suitable platform for integrating the germline-targeting features identified by neutralization.

### Cryo-EM structure of CH235 UCA2 bound to stabilized CH505TF.N279K.G458Y.SOSIP.664 Env

**Fig 3:**
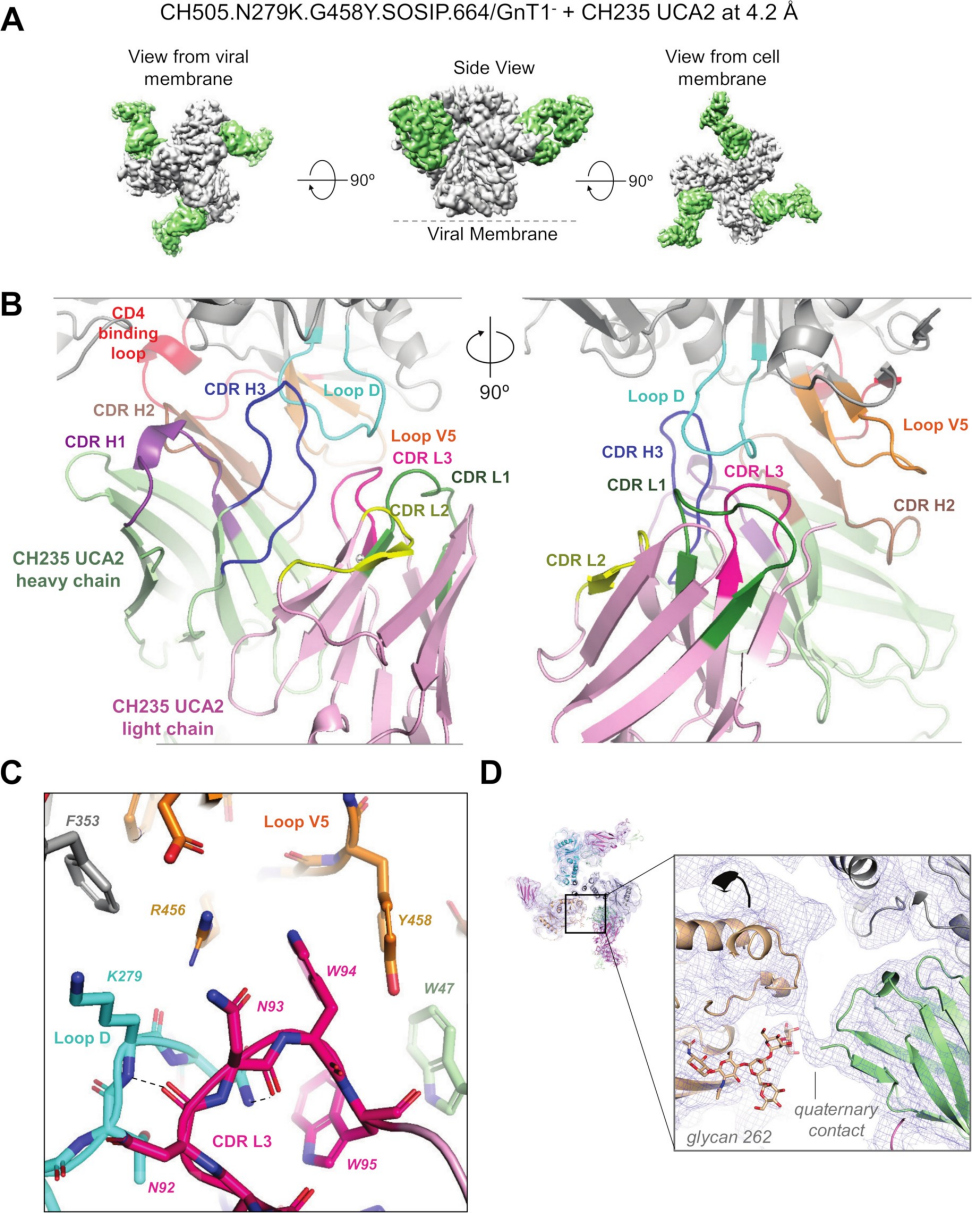
Cryo-EM structural details for reconstructions of CH235UCA complex with HIV-1 Env trimer CH505TF.N279K.G458Y.SOSIP.664. (A) Cryo-EM reconstruction of CH235 UCA2 bound to stabilized CH505TF.N279K.G458Y.SOSIP.664/GnT1^-^ trimer, shown segmented by component with CH235 UCA2 colored green and HIV-1 Env colored gray. (B) Zoomed-in view of the CH235 UCA2 binding interface with Env. The Env CD4 binding loop is shown in red, loop D in cyan, and loop V5 in orange. The CH235 UCA2 heavy chain is colored green, with the CHR H1, CDR H2 and CDR H3 loops colored purple, brown and blue, respectively. The CH235 UCA2 light chain is colored pink, with the CDR L1, CDR L2 and CDR L3 loops colored green, yellow, and magenta, respectively. The view on the right is rotated 90° clockwise with respect to the view shown on the left. (C) Zoomed-in view showing the interactions of the CH235 UCA2 CDR L3 (magenta) with the HIV-1 Env loop D (cyan) and loop V5 (orange). CDR H2 is shown in brown. The interactive residues are shown in stick representation and the black dotted lines indicate hydrogen bond interactions. (D) Left, cryo-EM reconstruction of the CH235 UCA2-Env complex shown in blue mesh with underlying fitted model shown in cartoon representation. Right, Zoomed-in view showing a quaternary interaction of the CH235 UCA2 heavy chain (green) N terminus with the adjacent protomer (wheat). Glycan 262 from the adjacent protomer is shown in stick representation.

To understand the structural basis for CH235 UCA2 recognition of Env with the N279K and G458Y mutations, we determined the structure of CH235 UCA2 in complex with Man_5_-enriched stabilized CH505TF.N279K.G458Y.SOSIP.664 by cryogenic electron microscopy (cryo-EM) (Fig 3, S4 and S5 Figs, S3 Table).

Reference-free 2D classification of particles picked from a total of 1,464 frame-aligned micrographs revealed classes that corresponded to Fab-bound trimeric Env, as well as classes that corresponded to smaller Fab-bound species, presumably Fab-bound monomeric gp120 (S4 Fig). An *ab initio* reconstruction generated from the former population revealed three Fabs bound symmetrically around the Env trimer. 3D refinement of the *ab initio* model against the cleaned-up stack of particles, without applying symmetry, confirmed three Fabs bound to the HIV-1 Env, following which 3-fold symmetry was applied to obtain the final reconstruction which refined to an overall resolution of 4.2 Å (Fig 3A, S4 Fig, S3 Table). The resolution of the cryo-EM reconstruction ranged from 4.0–8.5 Å, with the highest resolutions observed at the core of the complex and at the Env/antibody interface (S5A Fig).

The CH235 UCA2 Fab bound at the CD4 binding site, approaching each protomer at an angle similar to what was previously observed for structures of other CH235 lineage antibodies bound to monomeric gp120 (S6 Fig) [31]. Despite the modest resolution of the cryo-EM reconstruction, density was well-resolved for the antibody-gp120 interface (S5 Fig), allowing unambiguous placement of the complementarity determining regions (CDRs) of the antibody, as well as its epitope on the Man_5_-enriched CH505TF.N279K.G458Y.SOSIP.664 Env trimer.

The CH235 UCA2 Fab used its CDR H2 to mediate key contacts with the gp120 CD4 binding loop (Fig 3B), exhibiting the CD4 mimicry that is typical of the VRC01 and CH235/8ANC131 classes of CD4bs antibodies derived from the VH1-2 and VH1-46 heavy chain germline genes, respectively (S6 Fig) [46, 47, 48, 49]. The CH235 UCA2 also made extensive contacts with loop D and loop V5, which are the sites of the N279K and the G458Y mutations, respectively (Fig 3B and 3C, S5 and S6 Figs). CH235 UCA2 interfaced with loop D primarily via CDR H3 and CDR L3 (Fig 3B and 3C, S5B–S5E Fig). It also contacted the side chain of loop D K282 via residue Y33 in the CDR H1 loop (S5E Fig). The side chain of gp120 K279 adopted an extended conformation that allowed it to stack against the side chain of N93 in the CH235 UCA2 CDR L3 and form a van der Waals contact with the side chain of gp120 F353 (Fig 3C, S5D Fig). CH235 UCA2 CDR L3 made additional contacts with the loop D region around the engineered gp120 K279, via a hydrogen bond between the gp120 K279 backbone nitrogen and the UCA N92 backbone carbonyl oxygen, and two other hydrogen bonds between the gp120 N280 side chain and the UCA main chain carbonyls of residues N92 and N93. The UCA W95 side chain also interacted with the gp120 N280. The CH235 UCA2 CDR L3 appeared locked in place by its interactions with loop D and loop V5, with UCA W94 sequestered by cation-pi and pi-stacking interactions mediated by loop V5 residues R456 and Y458 (Fig 3C, S5B and S5C Fig). Apart from interacting with the CDR L3, the side chain of the engineered gp120 Y458 also stacked against the CDR H2 of CH235 UCA2 (Fig 3C, S5B and S5C Fig). We compared the structures of CH235 UCA2-bound Env determined in this study with that of gp120-bound CH235.12 determined previously using x-ray crystallography [31]. We found that the antibodies bound Env with overall similar angles of approach (S6A and S6B Fig). The interactions with key Env elements such as the CD4 binding loop, loop D and loop V5 were preserved. A change in conformation was observed in loop V5 to accommodate the bulky tyrosine side chain from the G458Y substitution (S5 and S6 Fig).

In addition to the N279K and G458Y mutations, Man_5_-enrichment of the CH505TF.N279K.G458Y.SOSIP.664 trimer enhanced binding to CH235 UCA2. In the cryo-EM reconstruction, although we observed densities for a number of glycan moieties proximal to the CD4 binding site for most sites, the map was not resolved well enough to fit and refine glycan models with correct stereochemistry. Glycan 262, however, showed reasonably well-resolved density that we were able to fit. We observed that glycan 262 from the adjacent protomer showed well defined density approaching the bound CH235 UCA2. To gain insights into the effect of Man_5_ enrichment, we aligned the CH235 UCA2 bound structure with that of HIV-1 clade A BG505 SOSIP.664 prefusion Env trimer in complex with the bnAbs PGT122 and 35022 (PDB ID:5FYL), where the SOSIP was expressed in 293F cells, thus resulting in complex type glycans. We found that glycans 301 and 262 from the quaternary protomer, when in complex form, could potentially obstruct the path of approach of CH235 UCA2 (S6I and S6J Fig). We also observed a quaternary contact in the cryo-EM density between the N terminus of the CH235 UCA2 heavy chain and the quaternary site of CD4 interaction on the Env trimer (Fig 3D) [50]. In summary, the cryo-EM structure provides atomic level insights into how the G458Y and N279K mutations permit high affinity binding between the engineered Man_5_-enriched CH505TF.N279K.G458Y.SOSIP.664 and CH235 UCA2.

**Fig 4:**
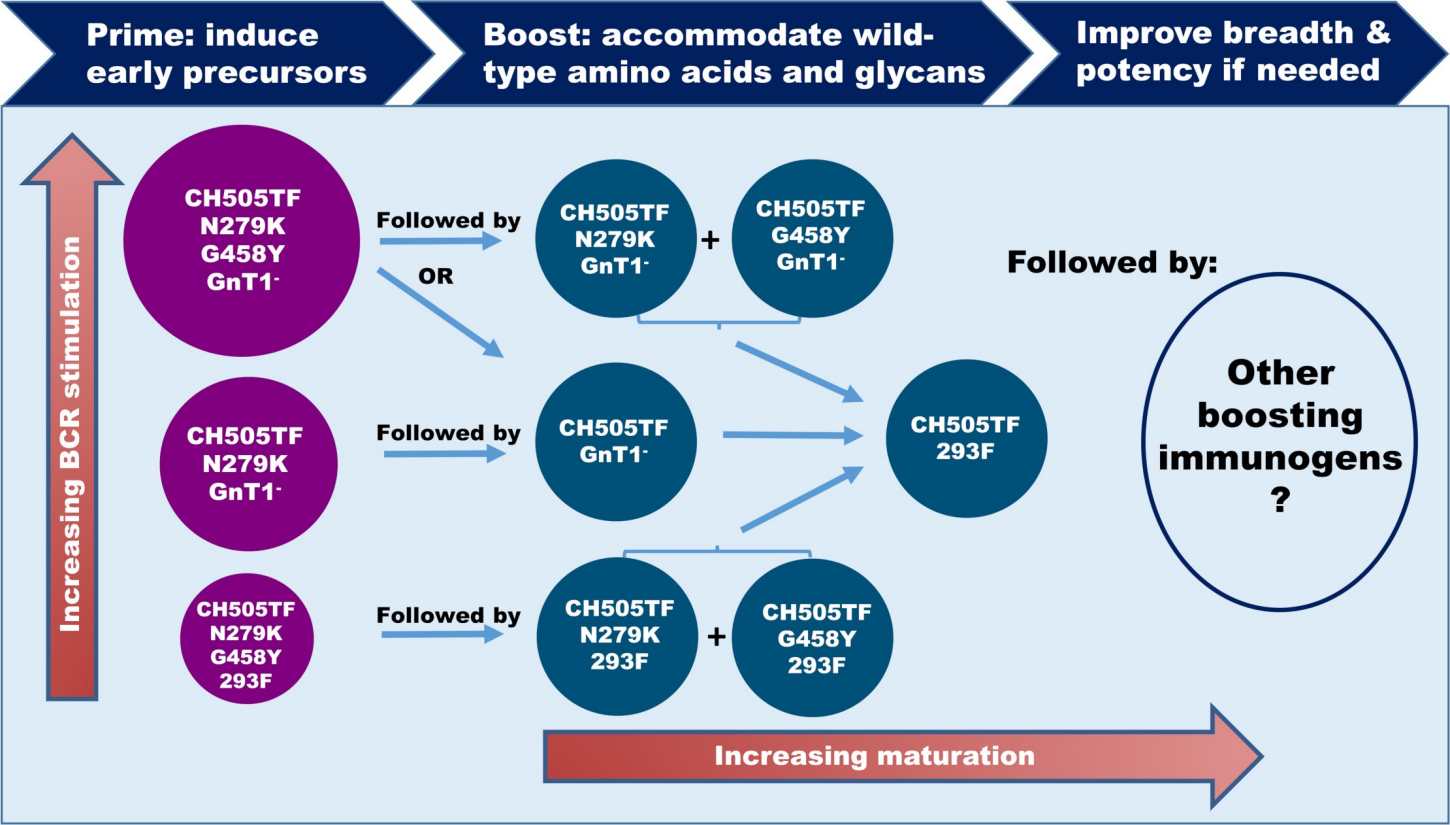
Immunization strategies to initiate and mature CH235 lineage bnAbs. A strategy is presented to elicit CH235 lineage bnAbs by first priming with an immunogen that is predicted to engage appropriate naïve B cells with high, medium or low relative affinities. Subsequent boosting immunogens would aim to mature the response by driving the somatic mutations required to accommodate N279, G458 and a fully processed glycan shield. The choice of boosting immunogens depends in part on which priming immunogen proves to be optimal for precursor induction. Man_5_-enriched immunogens would be produced in GnT1^-^ cells, and fully glycosylated immunogens would be produced in 293F or equivalent cells, where the cells for both immunogens are approved for GMP manufacturing.

The combination of the neutralization data and structural data provide strong support for a stable and functionally relevant interaction between the inferred germline antibody CH235 UCA2 and the CH505TF.N279K.G458Y Env. This leads us to hypothesize that immunization priming with the conformationally stable SOSIP.664 protein of this Env would stimulate naïve B cells bearing the germline antibody for the CH235 bnAb lineage. Indeed, the germline-targeting CH505TF.N279K.G458Y.SOSIP.664/GnT1^-^ immunogen induced CH235 early precursors in CH235 UCA2 knock-in mice, overcoming an improbable K19T heavy chain mutation, and induced a neutralization signature consistent with CH235 early precursors in rhesus macaques (Saunders et al., submitted). As shown in Fig 4 and discussed below, immunogens bearing various combinations of N279K, G458Y and/or Man_5_-enrichment offer potential prime-boost strategies to determine the most effective maturation pathway from early precursor to mature bnAb.

### Utility for immune monitoring

The ability to detect UCAs and intermediates of CH103 and CH235/CH235.12 in neutralization assays has utility for immune monitoring in pre-clinical and clinical trials. Based on our results, CH505TF.N279K.G458Y/GnT1^-^ is predicted to be highly sensitive for detecting vaccine-elicited early precursors of the CH235/CH235.12 lineage, where samples testing positive may be assayed against the N280D knock-out to confirm CD4bs-specificity (Fig 1, S1H and S1I Fig). Assays with 239T-grown mutant viruses and parental CH505TF/GnT1^-^ could be used to confirm a requirement for Man_5_-enrichment and the two mutations, respectively, as evidence for the complete neutralization signature seen with CH235 UCA2. One caveat is that the 293T version of the double mutant exhibits a tier 1A phenotype, making it susceptible to easily-induced antibodies that bind an open trimer conformation and are commonly induced by Env immunogens. This will need to be considered when interpreting results with this virus produced in 293T cells. Fortunately, the GnT1^-^ version of the virus exhibits a tier 2 phenotype, which is not susceptible to these easily induced antibodies and makes the virus more selective for antibodies of greater interest.

CH505TF.gly4/GnT1^-^ is predicted to be highly sensitive for detecting vaccine-elicited early precursors of CH103 (Fig 1). Confirmation of CD4bs specificity is not possible with N280D and other known CD4bs bnAb resistance mutations because these mutations did not impart resistance to CH103 and its intermediates. To design a suitable epitope knock-out virus, additional point mutations were investigated that in crystal structures are contacts for CH103 but not CD4 (to maintain infectivity). Three mutations in V5 (N461A, N462A and T463A) had no effect but a fourth mutation in the CD4-binding loop (S365P) conferred resistance to the UCA and intermediates while not conferring resistance to mature CH103 (S4 Table). With this knock-out mutant, we now have sets of viruses that can detect UCAs and intermediates of CH103 and CH235/CH235.12, identify intermediate stages and confirm epitope specificity with selectively resistant viruses. These will be valuable tools with which to evaluate antibody progression in pre-clinical and clinical trials aimed at eliciting CH103- and CH235-like bnAbs.

## Discussion

Env-pseudotyped virus neutralization is becoming a powerful tool to guide the design of bnAb lineage-based HIV-1 vaccines [29, 51]. Here, virus neutralization guided the identification of a vaccine strategy aiming to elicit the CH103 and CH235/CH235.12 lineages of CD4bs bnAbs. Engineering features were sought that render autologous CH505TF virus sensitive to neutralization by germline-reverted and early intermediates of CH103 and CH235/CH235.12, where neutralization served as a surrogate for native Env binding. Unlike the features that rendered the 426c virus sensitive to neutralization by germline-reverted VRC01-class bnAbs [29], glycan deletion combined with Man_5_-enrichment did not overcome the barrier for neutralization by CH235 UCA2. Nonetheless, the combination of glycan-deletion and Man_5_-enrichment was partially successful for CH103 UCA, and glycan deletion alone was largely successful for all intermediates of CH103 except the earliest intermediate tested (Fig 1).

The barrier to neutralization by CH235 UCA2 was overcome by synergistic interactions between two Env mutations, one in loop D (N279K, which does not remove an *N*-glycan) and another in V5 (G458Y). Additional potent synergy was provided by Man_5_-enrichment. N279K and G458Y confer resistance to VRC01-class bnAbs, such as VRC01 and 3BNC117, but do not impart resistance to mature CH103 and CH235/CH235.12 in the context of CH505TF (Fig 1). Previous structural studies have shown that early members of the CH235-lineage recognize regions that encompass both the invariant CD4 supersite of vulnerability, as well as regions that are outside this supersite and include the gp120 loops D and V5 [48]. Somatic hypermutation focuses recognition of the more mature antibodies of the lineage on the CD4 supersite of vulnerability [31]. Consistent with these previous findings, we observed a large interactive surface of the CH235 UCA2 on loops D and V5, with the mutated Env residues at gp120 positions 279 and 458 directly involved in interactions with the antibody. The cryo-EM structure revealed a central role for the CH235 UCA CDR L3 loop in recognizing both the engineered gp120 loops D and V5 (Fig 3C, S5B Fig). Interactions between the side chain of the gp120 loop D residue K279 with the CH235 UCA2 CDR L3, as well as with other gp120 regions (Fig 3C, S5C Fig), suggested a role for the N279K mutation in stabilizing the loop D structure and enabling its interactions with the CH235 UCA2 CDR L3. Similarly, the gp120 loop V5 G458Y mutation replaced the small Gly with a bulky Tyr side chain and enabled van der Waals interactions with the CH235 UCA2 CDR L3 residue W94 (Fig 3C, S5B and S5C Fig). These structural results show how the N279K and the G458Y mutations synergize to stabilize Env interactions with the CDR L3 loop of CH235 UCA2, and enable the binding of CH235 UCA2 to the CH505TF.N279K.G458Y Env.

We also found that the magnitude of virus neutralization by CH235 UCA2 correlated with binding affinities to corresponding CH505TF SOSIP trimers (Fig 2), indicating that SOSIP trimers may be a suitable platform for translating our neutralization-guided findings into viable immunogens. Fig 4 illustrates how these immunogens may be used in a germline-targeting and reverse engineering strategy to initiate and mature the CH235 bnAb lineage. CH505TF SOSIP trimers that trigger different levels of B cell activation afford opportunities to define optimal priming conditions to initiate the lineage. Boosting with reverse-engineered immunogens aims to train the B cells to accommodate wild-type amino acids at positions 279 and 458 and a fully processed glycan shield. Natural heterogeneity in the composition of glycans at occupied sites [52, 53] may facilitate the final accommodation of a “fully processed” glycan shield.

The fact that N279K does not remove an *N-*glycan reduces the possibility of eliciting a strain-specific glycan-hole response to this site.

Iterative testing of different combinations of engineered CH505TF SOSIP trimers may ultimately lead to the identification of a successful regimen. In support of this strategy, CH505.N279K.G458Y SOSIP/GnT1^-^, which has 9 nM affinity for CH235 UCA2 (Fig 2, S3 Fig), induces CH235 early precursors in CH235 UCA2 knock-in mice, overcoming an improbable K19T heavy chain mutation; whereas CH505.N279K gp120, which has low affinity for CH235 UCA2, did not induce these precursors (Saunders et al., submitted). Additional studies are underway that aim to extend the maturation of this response in mice and to translate this strategy to higher order animal models. Progress in these and other immunization strategies aiming to elicit the CH235 bnAb lineage can be monitored in neutralization assays with engineered CH505TF Env-pseudotyped viruses that detect early precursors. Of note, these immunogens may also generate off-target responses, such as antibodies against exposed epitopes on open trimers [40], much the same as in HIV-1-infected individuals, including the source donor of CH103 and CH235 [30]. Whether and how these off target responses will impact the ability to elicit bnAbs by vaccination is unknown.

In summary, Env-pseudotyped virus neutralization permitted the identification of two Env mutations that acted synergistically to render CH505TF highly sensitive to neutralization by CH235 UCA2, with additional synergy provided by Man_5_-enriched *N*-glycans. Neutralization potency correlated with nanomolar affinity binding to corresponding SOSIP trimers as candidate immunogens. These findings suggest a lineage-based vaccine approach aimed at eliciting CH235/CH235.12-like bnAbs and provide a neutralization-based strategy for monitoring vaccine-elicited early precursors of this bnAb lineage.

## Methods

### Ethics statement

This study utilized pre-existing, de-identified human serum samples obtained from the Duke Center for HIV Vaccine Immunology (CHAVI) repository under approval of the Duke University Health System Institutional Review Board (Pro00015593). The data were analyzed anonymously.

### Cells

TZM-bl, HEK 293T/17 and HEK 293S/GnT1^-^ cells were maintained in Dulbecco's Modified Eagle's Medium (DMEM) containing 10% fetal bovine serum (FBS) and gentamicin (50 μg/ml) in vented T-75 culture flasks (Corning-Costar). Cultures were incubated at 37°C in a humidified 5% CO2–95% air environment. Cell monolayers were split 1:10 at confluence by treatment with 0.25% trypsin, 1 mM EDTA.

### Antibodies and HIV-1 sera

VRC01 [54], VRC34.01 [55] and 10E8 [56] were produced by the Vaccine Research Center, NIH. N6 [57] was obtained from Dr. Mark Connors. 3BNC117 [58] and 10–1074 [59] were obtained from Dr. Michel Nussenzweig. VRC-CH31 and CH01 [60] were produced by Catalent Biologics (Madison, WI). DH511.2_K3 [61] was produced by the Human Vaccine Institute, Duke University Medical Center. HJ16 [62] was obtained from Dr. Davide Corti. B12 [63], 2G12, 2F5, 4E10, PG9 and PG16 were purchased from Polymun Scientific (Klosterneuburg, Austria). PGDM1400 [64], PGT121 [65], PGT128 [65] and PGT151 [35] were a kind gift from Dr. Dennis Burton. VRC01, 3BNC117, VRC-CH31 and N6 belong to the VRC01-class of bnAbs characterized by heavy-chain mimicry of the CD4 receptor, VH1-2 germline gene usage, and a 5-amino acid CDRL3.

In addition to these mature bnAbs, we utilized UCAs, intermediates and mature forms of CH103 and CH235/CH235.12 [31, 32], which were produced by the Human Vaccine Institute, Duke University Medical Center, Durham, North Carolina. The unmutated common ancestor (UCA) sequence for the CH235/CH235.12 lineage used in this study differs by one amino acid from the UCA described previously [31]. The UCA used here, which we refer to as CH235 UCA2, has a methionine in the 4th position of the light chain in place of a leucine in the previously described UCA version. We also included a germline-reverted form of VRC01 [46], which was produced at the Vaccine Research Center, NIH. This latter germline-reverted antibody possess mature HCDR3 and J regions whose germlines could not be inferred with existing sequence information. Heavy and light chain sequences of the germline-reverted and intermediate antibodies tested here may found in LaBranche et al. [29].

Neutralization tier phenotyping was performed with serum pools from individuals in southern Africa (South Africa, Malawi and Tanzania) who participated in a CHAVI study of chronic HIV-1 infection (CHAVI samples 0293, 0537, 0642, 0461 and 0598). These study subjects had all been infected for at least three years. Samples from 6–10 time points collected over 8–60 months were pooled on a per-subject basis and heat-inactivated for 30 minutes at 56°C. For deeper interrogation of neutralization phenotype, a set of monoclonal antibodies that show a strong preference for tier 1 viruses was used. This set included V3-specific antibodies 2219 [66], 2557 [67], 3074 [68], 3869 [69], 447-52D [70] and 838-D [71], and the CD4bs antibodies 654-30D [72], 1008-30D [73], 1570D [73] and 729-30D [74] produced by Drs. Susan Zolla-Pazner and Miroslaw K. Gorny at New York University and the Veterans Affairs Medical Center, New York, New York. It also included the CD4bs antibody F105 [75] produced by the Protein Production Facility in the Duke Human Vaccine Institute.

### Pseudotyping Envs

Full-length functional HIV-1 Envs were used for virus pseudotyping. Previous reports described Envs for strains CH505TF and CH505.w4.3 [31]. Glycan deleted Envs CH505TF.gly4, CH505TF.gly197, CH505TF.gly3.276 and CH505TF.gly3.461 were described previously [33]. In some cases additional mutations introduced by site-directed mutagenesis as described [76].

### Transfection

Env-pseudotyped viruses were produced in either 293T/17 or 293S GnT1^-^ cells (American Type Culture Collection) as described [77]. 293S GnT1^-^ cells lack the enzyme *N-*acetylglucosaminyltransferase and have been shown to yield HIV-1 Envs that contain Man_6-9_ glycoforms and are enriched for under-processed Man_5_ glycoforms in place of complex glycans [37, 78]. Env-pseudotyped viruses were generated by transfecting exponentially dividing 293T/17 or 293S GnT1^-^ cells (5 X 10^6^ cells in 12 ml growth medium in a T-75 culture flask) with 4 μg of rev/env expression plasmid and 8 μg of an env-deficient HIV-1 backbone vector (pSG3ΔEnv), using Fugene 6 transfection reagent. Cells were washed after 3–8 hours and incubated in fresh growth medium without transfection reagents. Env-pseudotyped virus-containing culture supernatants were harvested 2 days after transfection, filtered (0.45 μm), and stored at -80°C in 1 ml aliquots. Infectivity was quantified in TZM-bl cells by performing serial fivefold dilutions of pseudovirus in quadruplicate wells in 96-well culture plates in a total volume of 100 μl of growth medium for a total of 11 dilution steps. Freshly trypsinized cells (10,000 cells in 100 μl of growth medium containing 75 μg/ml DEAE-dextran) were added to each well, and the plates were incubated at 37°C in a humidified 5% CO_2_−95% air environment. After a 48-hour incubation, 100 μl of culture medium was removed from each well and 100 μl of Britelite reagent was added to the cells. After a 2-min incubation at room temperature to allow cell lysis, 150 μl of cell lysate was transferred to 96-well black solid plates (Corning-Costar) for measurements of luminescence using a Victor 3 luminometer (Perkin-Elmer Life Sciences, Shelton, CT). A dilution of virus that results in 50,000–250,000 relative luminescence units (RLUs) was used for neutralization assays.

### Neutralization assay

Neutralization assays were performed in TZM-bl cells (NIH AIDS Research and Reference Reagent Program, contributed by John Kappes and Xiaoyun Wu) as described [77]. Briefly, a pre-titrated dose of Env-pseudotyped virus was incubated with serial 3-fold or 5-fold dilutions of test sample in duplicate in a total volume of 150 μl for 1 hr at 37°C in 96-well flat-bottom culture plates. Freshly trypsinized cells (10,000 cells in 100 μl of growth medium containing 20 μg/ml DEAE dextran) were added to each well. One set of control wells received cells + virus (virus control) and another set received cells only (background control). After 48 hours of incubation, the cells were lysed by the addition of Britelite (PerkinElmer Life Sciences) and three quarters of the cell lysate was transferred to a 96-well black solid plate (Costar) for measurement of luminescence. Neutralization titers are either the serum dilution (ID50) or antibody concentration (IC50) at which relative luminescence units (RLU) were reduced by 50% compared to virus control wells after subtraction of background RLUs. For all reported results, the average RLU of virus control wells was >10 times the average RLU of cell control wells, the percent coefficient of variation (%CV) between RLU in the virus control wells was ≤30%, the percent difference between duplicate wells was ≤30%, and neutralization curves cross the 50% neutralization cut-off 0–1 times. Unless otherwise stated or illustrated, the values and neutralization curves are from a single assay (duplicate wells).

### Design of stabilized HIV-1 SOSIP gp140s

CH505TF.N279K.G458Y.SOSIP.664 was stabilized using a BG505 chimeric design where the CH505TF gp120 was grafted into the BG505 SOSIP gp140 sequence resulting in 29 amino acid changes from the parental CH505TF sequence [33, 45]. Additionally, E64K and A316W mutations were introduced into the gp120 to stabilize the pre-CD4 bound conformation and prevent V3 loop exposure [23, 45]. N279K and G458Y mutations were added to this SOSIP design in various combinations.

### Recombinant HIV-1 gp140 SOSIP production

SOSIP gp140s were expressed, purified and characterized as previously described with minor modifications [45]. SOSIP production was performed with Freestyle293 (293F) cells or 293S GnT1^-^ cells to produce SOSIPs with heterogeneous or Man_5_GlcNac_2_-enriched glycosylation, respectively. Prior to transfection, the 293 GnT1^-^ cells were cultured in ESF SFM (Expression Systems) media mixed in equal volume with Freestyle293 media. On the day of transfection, Freestyle293 and 293S GnT1^-^ cells were diluted to 1.25x10^6^ cells/mL with fresh Freestyle293 media up to 1L total volume. The cells were co-transfected with plasmid DNA complexed with 293Fectin. For each 1L transfection, 650 μg of SOSIP expressing plasmid and 150 μg of furin expressing plasmid were used. On day 6, cell culture supernatants were harvested by centrifugation of the cell culture for 30 min at 3500 rpm. The cell-free supernatant was filtered through a 0.8 μm filter and concentrated to less than 100 mL with a single-use tangential flow filtration cassettes and 0.8 μm filtered again. SOSIP protein was purified with PGT145 affinity chromatography. One hundred mg of PGT145 IgG1 antibody was conjugated to 10 mL of CnBr-activated sepharose FastFlow resin (GE Healthcare). Coupled resin was packed into Tricorn column (GE Healthcare), and stored in PBS supplemented with 0.05% sodium azide. Cell-free supernatant was applied to the column at 2 mL/min using an AKTA Pure (GE Healthcare), washed, and protein was eluted off the column with 3M MgCl_2_. The eluate was immediately diluted in 10 mM Tris pH 8, 0.2 μm filtered, and concentrated down to 2 mL for size exclusion chromatography. Size exclusion chromatography was performed with a Superose6 16/600 column (GE Healthcare) in 10 mM Tris pH 8, 500 mM NaCl to identify trimeric protein. Fractions containing trimeric HIV-1 Env protein were pooled together, sterile-filtered, snap frozen, and stored at -80°C.

The formation of trimers was determined by negative stain electron microscopy (S4 Fig). Unbiased two-dimensional class averages were generated with EMAN2 [45]. Trimer formation was also confirmed by blue native-PAGE and analytical size exclusion chromatography. Antigenicity of the trimers in solution were determined by biolayer interferometry (S4 Fig).

### Surface plasmon resonance (SPR)

The rate constants and the apparent dissociation rate constants (K_D_) of CH235 UCA2 binding to trimeric SOSIP proteins was performed by surface plasmon resonance (SPR) using the Biacore 3000 (GE Healthcare) as described previously [79]. A CM3 sensor chip was used to directly immobilize approximately 4500 RU (response units) of a goat Anti-Human IgG Fc antibody (Millimore/Sigma, Bedford, MA). The Anti-Human IgG Fc was then used to capture the CH235 UCA2 antibody to a level of 200-350RU. Proteins were diluted from 50nM-750nM in HBS-EP+ buffer (GE Healthcare) and then injected over the antibody captured surfaces for 5 minutes at 50 ul/min. The 5 minute analyte injection was followed by a 10 minute dissociation period with buffer wash and then a 20 second injection pulse of Glycine pH 2.0 for regeneration. Kinetics results were analyzed using the BIAevaluation 4.1 software (GE Healthcare). A negative control antibody, Ab82 (Anti-influenza hemagglutinin, Catalant), and buffer binding were used for double reference subtraction to account for non-specific binding and signal drift. Subsequent curve fitting analysis was performed using a 1:1 Langmuir model with a local Rmax and the reported rate constants are representative of 2 measurements.

### Cryo-EM data collection and processing

Env trimer used in cryo-EM was generated in GnT1^-^ cell line as described above. To prepare Env complexes, CH505TF.N279K.G458Y.SOSIP.664 at a final concentration of 1 mg/ml was incubated with 4–5–fold molar excess of the CH235 UCA2 Fab fragments for 30–60 minutes. To prevent aggregation during vitrification, the sample was incubated in 0.085 mM dodecyl-maltoside (DDM). The specimen was vitrified by applying 2.5 μl of sample to freshly plasma-cleaned Quantifoil holey carbon grids (1.2/1.3–3C), allowing the sample to adsorb to the grid for 60 s, followed by blotting with filter paper and plunge-freezing into liquid ethane using the Leica EM GP cryo-plunger (Leica Microsystems) (20°C, >90% relative humidity). Data were acquired using the EPU automated data collection software installed on a Titan Krios transmission electron microscope operating at 300kV and fitted with Falcon3 Direct Electron Detector operating in counting mode. The dose was fractionated over 30 raw frames and collected over 60 s with an exposure of ~0.8 e^-^/pixel/s for a total exposure of ~42 e^-^/Å^2^. Individual frames were aligned and dose-weighted.

Contrast transfer function (CTF) was estimated using the GCTF package [80]. Particles were picked using the Laplacian-of-Gaussian function in RELION 3 [81]. These particles were imported into cryoSparc [82], where 2D classification was performed, and selected 2D classes representing different views of the complex were used for template-based particles picking. Following further 2D classifications to remove junk, *ab initio* reconstruction and classification was performed using C1 symmetry. An *ab initio* model with three antibody Fabs bound symmetrically to the HIV-1 Env trimer was identified, and was refined using C3 symmetry against the clean stack of particles to obtain a 4.2 Å resolution map. Overall map resolution was reported according to the FSC_0.143_ gold standard criterion.

### Cryo-EM model fitting

Fits of HIV-1 trimer and Fab to the cryo-EM reconstructed maps were performed using UCSF Chimera [83]. BG505 SOSIP trimer structure (PDB ID: 5YFL) was used for the trimer fits and the coordinates of mature CH235.12 (PDB ID: 5F96) were used for fitting the Fab. The sequences were replaced with those of the CH505TF.N279K.G458Y.SOSIP.664 trimer and CH235 UCA2, respectively using Coot [84]. The coordinates were further fit to the electron density first using Rosetta [85], followed by an iterative process of manual fitting using Coot and real space refinement within Phenix [86] with a final round of Rosetta refinement. Molprobity [87] and EMRinger [88] were used to check geometry and evaluate structures at each iteration step. Glycans were validated using pdb-care [89]. Figures were generated in UCSF Chimera and PyMOL (The PyMOL Molecular Graphics System, Version 2.0 Schrödinger, LLC). Map-fitting cross correlations were calculated using Fit-in-Map feature in UCSF Chimera. Map-to-model FSC curves were generated using EMAN2. Local resolution of cryo-EM maps was determined using RELION 3.

## Supporting information

S1 Table Infectivity of Env-pseudotyped viruses in TZM-bl cells. (PDF)

S2 Table Impact of amino acids other than lysine (K) at position 279 and tyrosine (Y) at position 458 of CH505TF Env. (PDF)

S3 Table Cryo-EM data collection and refinement statistics. (PDF)

S4 Table S365P is a resistance mutation for the UCA and intermediates of CH103. (PDF)

S1 Fig Neutralization curves for UCAs and intermediates of CH103 and CH235. Neutralization assays were performed with Env-pseudoyped viruses prepared in either 293T or 293S/GnT1^-^ cells and assayed in TZM-bl cells as described in Methods. The dilution factor (i.e., 3-fold or 5-fold) and range of bnAb concentrations evaluated varied depending on the potency of virus/antibody combinations, and sometimes multiple dilution factors and ranges were evaluated in repeat assays. This was done to obtain curves that were linear when crossing 50% neutralization for accurate measurement of IC50 values. When multiple assays were performed, the average IC50 was used in Fig 1. CH103_IA_9_4A is the earliest intermediate of this lineage tested while CH103_AI_4_4A is the latest. Likewise, CH235_I4_v2_4A is the earliest intermediate while CH235_I1_v2_4A is the latest intermediate tested. (A) CH103_UCA_4A; (B) CH103_IA_9_4A; (C) CH103_IA_8_4A; (D) CH103_IA_7_4A; (E) CH103_IA_6_4A; (F) CH103_IA_5_4A; (G) CH103_IA_4_4A; (H) CH235 UCA2; (I) CH235_I4_v2_4A; (J) CH235_I3_v2_4A; (K) CH235_I1_v2_4A.
(PDF)

S2 Fig Recombinant stabilized CH505 SOSIP gp140s form trimeric envelope and possess the antigenic profile of envelope in the closed conformation. (A) Representative two-dimensional class averages of negative stain electron microscopy images of CH505 SOSIP gp140 proteins. The amino acid changes introduced into the CH505 transmitted/founder envelope sequence are indicated above each picture. (B) Antigenicity of each CH505 SOSIP gp140 variant as determined by biolayer interferometry (BLI). Values are binding responses in nm. GnT1^-^ indicates proteins produced in 293S GnT1^-^ cells to enrich for Man_5_GlcNac_2_.
(PDF)

S3 Fig Kinetics of SPR binding of CH235 UCA2 to parental and mutant CH505TF SOSIP trimers produced in either 293F or 293S GnT1- cells. SPR affinity data are apparent K_D_ values with rate constants derived from curve fitting analyses to 1:1 Langmuir model for binding of trimeric SOSIP proteins as analyte to CH235 UCA2 mAb immobilized on anti-Ig Fc mAb on the sensor chip. Concentration of SOSIP proteins in each binding curve is given. Data in table shown are results of two independent measurements by SPR analyses.
(PDF)

S4 Fig Structural details for cryo-EM reconstruction of CH235 UCA2 bound to Man5-enriched CH505TF.N279K.G458Y.SOSIP.664. (A) Representative micrograph. (B) Initial 2D class averages showing larger complexes corresponding to Fab bound to Env trimer marked with red dots, and smaller complexes corresponding to a single Fab bound to an Env fragment, presumably gp120, marked with blue dots. (C) Final 2D class averages. (D) *Ab initio* model. (E) Refined map starting from *ab initio* generated model and refining it against a stack of cleaned-up particles, and applying C3 symmetry. (F) Fourier shell correlation curve. The dotted line indicates FSC_0.143_.
(PDF)

S5 Fig Local map resolution for cryo-EM reconstruction of CH235 UCA2 bound to Man5-enriched CH505TF.N279K.G458Y.SOSIP.664. (A) Local Resolution color coded and plotted on the map surface (B-E) Zoomed-in views of the Loop V5 and Loop D regions of HIV-1 Env gp120 and the bound CH235 UCA2 antibody. The blue mesh indicates experimental cryo-EM density, and the underlying fitted model is shown in cartoon and stick representation.
(PDF)

S6 Fig Comparison of the structure of CH235 UCA2 bound to Man5-enriched CH505TF.N279K.G458Y.SOSIP.664 with CH235.12 bound to HIV-1 clade A/E 93TH057 gp120 (PDB ID 5F96). (A and B) Overlay of gp120 outer domains (gray) to show angles of approach of CH235 UCA2 and CH235.12 antibodies. (C-H) Comparison of CH235 UCA2 and CH235.12 binding to critical elements on gp120, with the CD4 binding loop shown in C and D, loop D shown in E and F, and loop V5 shown in G and H. (I) Env-bound CH235 UCA2 structure. (J) Zoomed-in view showing an overlay of gp120 from PDB ID 5FYL on the quaternary protomer. The SOSIP trimer in the 5FYL crystal structure was produced in 293F cells. Glycans 301 and 262 from the quaternary protomer, when in complex form, show close approach to CH235 UCA2.
(PDF)
