# Correction: Neutralization-guided design of HIV-1 envelope trimers with high affinity for the unmutated common ancestor of CH235 lineage CD4bs broadly neutralizing antibodies

**DOI:** 10.1371/journal.ppat.1008200

**Published:** 2019-12-02

**Authors:** Celia C. LaBranche, Rory Henderson, Allen Hsu, Shay Behrens, Xuejun Chen, Tongqing Zhou, Kevin Wiehe, Kevin O. Saunders, S. Munir Alam, Mattia Bonsignori, Mario J. Borgnia, Quentin J. Sattentau, Amanda Eaton, Kelli Greene, Hongmei Gao, Hua-Xin Liao, Wilton B. Williams, James Peacock, Haili Tang, Lautaro G. Perez, Robert J. Edwards, Thomas B. Kepler, Bette T. Korber, Peter D. Kwong, John R. Mascola, Priyamvada Acharya, Barton F. Haynes, David C. Montefiori

The following information is missing from the Funding statement: M.J.B. and A.H. received funding from the Intramural Research Program of the NIH; National Institute of Environmental Health Sciences (ZIC ES103326 to M.J.B).

The Data Availability statement for this paper is incorrect. The correct statement is: The cryo-EM data of CH235 UCA bound to Man5-enriched CH505.N279K.G458Y.SOSIP.664 have been deposited to EMDB database with the accession code EMD-20735, and the fitted coordinates have been deposited to the RCSB database with accession number 6UDA.
