# Supplementary material for: Neutralization-guided design of HIV-1 envelope trimers with high affinity for the unmutated common ancestor of CH235 lineage CD4bs broadly neutralizing antibodies
Source: PLoS Pathog. 2019 Sep 17;15(9):e1008026. doi: 10.1371/journal.ppat.1008026 (PMC6764681; doi:10.1371/journal.ppat.1008026)
Supplement: S1 Table — (PDF) [file ppat.1008026.s001.pdf]

S1 Table. Infectivity of Env-pseudotyped viruses in TZM-bl cells.[1]

	Virus dilution yielding 150,000 RLU		Relative infectivity[2]		RLU (undiluted virus)[3]	
**Virus**	**293T**	**GnT1-**	**293T**	**GnT1-**	**293T**	**GnT1-**
CH505TF	499	47	1	1	ND[5]	1,324,808
CH505TF.gly4	175	25	0.35	0.53	2,061,808	1,088,156
CH505TF.gly3.197	853	45	1.71	0.96	ND	1,489,136
CH505TF.gly3.276	230	32	0.46	0.68	ND	1,165,652
CH505TF.gly3.461	179	10	0.36	0.21	1,984,298	879,184
CH505TF.G458Y	9	1	0.02	0.02	671,616	230,418
CH505TF.N279K	45	9	0.09	0.19	938,308	752,864
CH505TF.N279K.G458Y	<1[4]	<1[4]	<0.002	<0.02	145,732	19,726
CH505TF.N279K.G458Y.N280D	14	<1[4]	0.03	<0.02	759,184	119,464
CH505.w4.3	4	47	0.01	1	1,192,674	ND

[1]Env-pseudotyped viruses were produced by transfection in either 293T or 293S/GnT1- cells. TZM-bl cells were incubated with ten 5-fold dilutions of the virus in quadruplicate. Relative luminescence units (RLU) were measured after 2 days as described in Methods.

[2]Relative to CH505TF.

[3]Undiluted virus (50 ul) added to 200 ul of TZM-bl cells at the start of the dilution series.

[4]Undiluted virus yielded <150,000 RLU.

[5]ND, not determined because the dose resulted in extensive virus-induced cell killing.
