# Supplementary material for: Neutralization-guided design of HIV-1 envelope trimers with high affinity for the unmutated common ancestor of CH235 lineage CD4bs broadly neutralizing antibodies
Source: PLoS Pathog. 2019 Sep 17;15(9):e1008026. doi: 10.1371/journal.ppat.1008026 (PMC6764681; doi:10.1371/journal.ppat.1008026)
Supplement: S2 Table — (PDF) [file ppat.1008026.s002.pdf]

S2 Table. Impact of amino acids other than lysine (K) at position 279 and tyrosine (Y) at position 458 of CH505TF

TIER PHENOYTYPING (All viruses produced in GnT1$^-$ cells)

	ID50 (dilution) in TZM-bl cells						
Mutation in CH505TF.G458Y/GnT1-	CHAVI-0293pool2	CHAVI-0537pool2	CHAVI-0642pool2	CHAVI-0461pool2	CHAVI-0598pool2	GMT[1]	Tier
N279K	50	111	36	178	786	123	2
N279A	59	125	64	100	391	113	2
N279V	56	93	68	179	411	121	2
N279Y	47	99	61	68	253	87	2
N279W	62	79	69	127	402	112	2
N279S	34	54	27	47	293	58	2
N279T	110	155	90	257	8683	321	2
N279C	83	128	79	262	1905	211	2
N279M	143	256	123	261	2142	302	2
N279L	97	147	80	336	1244	217	2
N279R	47	76	35	77	432	84	2
N279H	<30	79	<30	70	476	57	2
N279D	54	121	47	90	608	111	2
N279E	143	308	131	333	2360	340	2
N279Q	55	127	60	180	327	120	2
Mutation in CH505TF/GnT1-							
G458Y	43	89	36	66	382	81	2
G458F	89	184	99	151	463	163	2
G458W	50	99	46	85	329	91	2
G458R	64	122	60	114	394	116	2
G458K	87	148	114	171	546	169	2
G458C	154	165	143	163	570	202	2
G458L	151	173	121	181	620	204	2
G458S	125	100	69	74	454	124	2
G458D	391	494	356	651	1705	598	1B
G458E	96	106	75	64	598	124	2
CH0505TF.N279K.G458C	1032	271	469	1846	1244	787	1B
CH0505TF.N279K.G458L	1893	891	883	672	1197	1037	1B
G458Y.N280D	30	35	<30	<30	162	33	2

CH235 LINEAGE PHENOTYPING (All viruses produced in GnT1$^-$ cells)

	IC50 (µg/ml) in TZM-bl cells				
Mutation in CH505TF.G458Y/GnT1-	CH235 UCA2	CH235_I4_v2_4A	CH235_I3_v2_4A	CH235VH_I1_v2_4A	CH235
N279K	0.02	0.05	0.011	0.003	0.002
N279A	8.46	0.05	0.009	0.004	0.002
N279V	26	0.04	0.008	0.007	0.002
N279Y	0.04	0.011	0.011	0.008	0.002
N279W	0.22	0.02	0.007	0.004	0.001
N279S	>50	0.16	0.001	0.007	0.001
N279T	4.35	0.02	0.009	0.007	0.003
N279C	1.38	0.04	0.018	0.008	0.005
N279M	0.12	0.005	0.005	0.005	0.002
N279L	0.23	0.018	0.009	0.008	0.003
N279R	0.05	0.004	0.006	0.003	0.001
N279H	1.26	0.034	0.013	0.011	0.002
N279D	>50	>50	0.068	0.036	0.005
N279E	>50	0.71	0.032	0.014	0.004
N279Q	0.62	0.004	0.007	0.006	0.006
Mutation in CH505TF/GnT1-					
G458Y	35.5	0.08	0.03	0.012	0.011
G458F	>50	0.720	0.020	0.020	0.003
G458W	>50	0.340	0.009	0.008	0.002
G458R	>10	0.470	0.025	0.013	0.009
G458K	>50	2.180	0.074	0.026	0.022
G458C	6.390	0.017	0.040	0.015	0.009
G458L	8.660	0.150	0.033	0.018	0.015
G458S	>50	2.980	0.042	0.014	0.005
G458D	>50	>50	0.063	0.005	0.011
G458E	>50	>50	0.074	0.020	0.012
CH0505TF.N279K.G458C	0.018	0.006	0.015	0.006	0.001
CH0505TF.N279K.G458L	0.013	0.002	0.011	0.006	0.0017
G458Y.N280D	>50	>50	7.820	0.070	2.691

[1]GMT, geometric mean titer

Weaker Stronger

Key:

Tier Classification	Range (ID50)
1A	>2000
1B	350 - 2000
2	50 - 350
3	<50
