# Supplementary material for: Neutralization-guided design of HIV-1 envelope trimers with high affinity for the unmutated common ancestor of CH235 lineage CD4bs broadly neutralizing antibodies
Source: PLoS Pathog. 2019 Sep 17;15(9):e1008026. doi: 10.1371/journal.ppat.1008026 (PMC6764681; doi:10.1371/journal.ppat.1008026)
Supplement: S3 Table — (PDF) [file ppat.1008026.s003.pdf]

**S3 Table. Cryo-EM data collection and refinement statistics**

| | CH505.N279K.G458Y.SOSIP.664 +CH235 UCA2 |
|---|---|
| Data Collection | |
| Microscope | FEI Titan Krios |
| Voltage (kV) | 300 |
| Electron dose ($e^-/Å^2$) | 42 |
| Detector | Falcon 3 |
| Pixel Size (Å) | 1.08 |
| Defocus Range (µm) | ~1.5-3 |
| Magnification | 75000 |
| | |
| Reconstruction | |
| Software | cryoSparc |
| Particles | 22093 |
| Symmetry | C3 |
| Box size (pix) | 320 |
| Resolution (Å) (FSC0.143)[$] | 4.2 |
| | |
| Refinement (Phenix) | |
| Protein residues | 3054 |
| Chimera CC | 0.85 |
| R.m.s. deviations | |
| Bond lengths (Å) | 0.006 |
| Bond angles (°) | 0.946 |
| | |
| Validation | |
| Molprobity score | 1.0 |
| Clash score | 1.4 |
| Favored rotamers (%) | 99.0 |
| Ramachandran | |
| Favored regions (%) | 97.1 |
| Disallowed regions (%) | 0.5 |
| EMRinger score | 1.47 |

[1]Resolutions are reported according to the FSC 0.143 gold-standard criterion
