# Supplementary material for: Neutralization-guided design of HIV-1 envelope trimers with high affinity for the unmutated common ancestor of CH235 lineage CD4bs broadly neutralizing antibodies
Source: PLoS Pathog. 2019 Sep 17;15(9):e1008026. doi: 10.1371/journal.ppat.1008026 (PMC6764681; doi:10.1371/journal.ppat.1008026)
Supplement: S4 Table — (PDF) [file ppat.1008026.s004.pdf]

S4 Table. S365P is a resistance mutation for the UCA and intermediates of CH103.

	**IC50 (μg/ml) in TZM-bl cells**	
**Antibody Name**	**CH505TF.gly4 293S GnT1$^-$**	**CH505TF.gly4.S365P 293S GnT1$^-$**
CH103_UCA_4A/293i	2.5	>50
CH103_IA_9_4A	>50	>50
CH103_IA_8_4A	0.002	>5
CH103_IA_7_4A	0.001	0.54
CH103_IA_6_4A	0.001	0.59
CH103_IA_5_4A	0.002	>5
CH103_IA_4_4A	0.001	0.817
CH103 (Mature)	0.002	0.002
CH235.12	0.002	0.002
