# Supplementary material for: Neutralization-guided design of HIV-1 envelope trimers with high affinity for the unmutated common ancestor of CH235 lineage CD4bs broadly neutralizing antibodies
Source: PLoS Pathog. 2019 Sep 17;15(9):e1008026. doi: 10.1371/journal.ppat.1008026 (PMC6764681; doi:10.1371/journal.ppat.1008026)

S1A Fig. Neutralization by CH103_UCA_4A.

293T

GnT1-

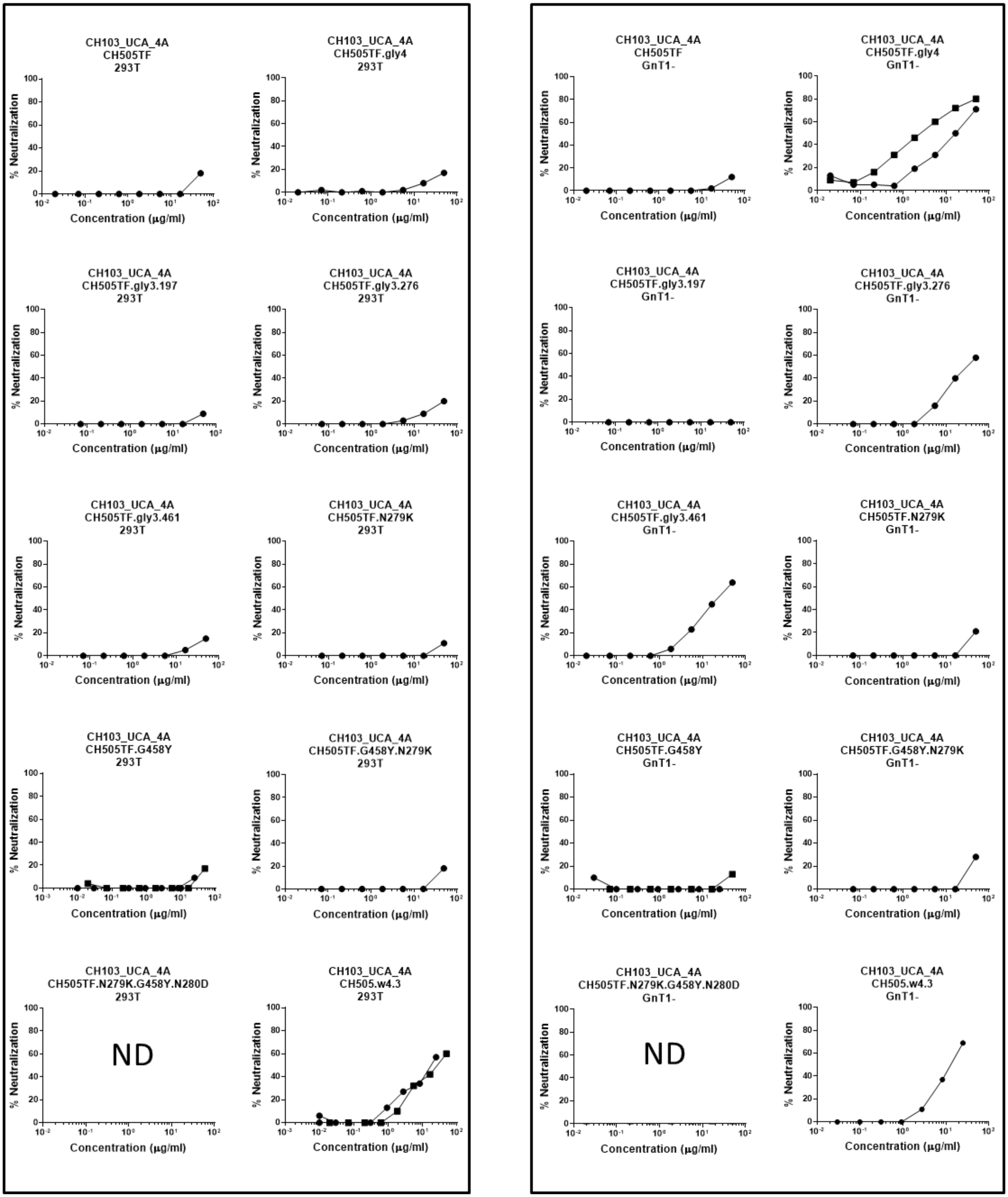

S1B Fig. Neutralization by CH103_IA_9_4A.

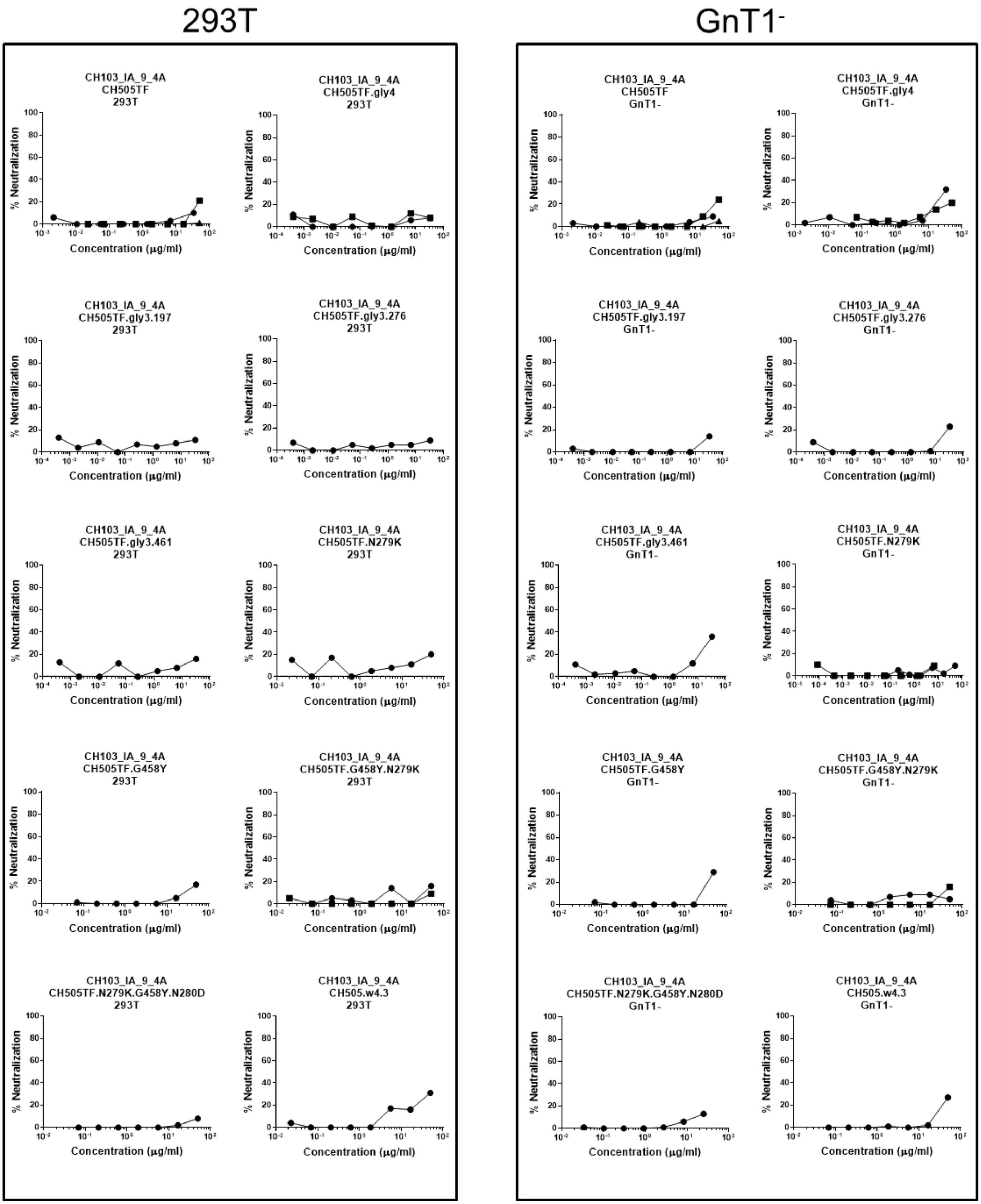

S1C Fig. Neutralization by CH103_IA_8_4A.

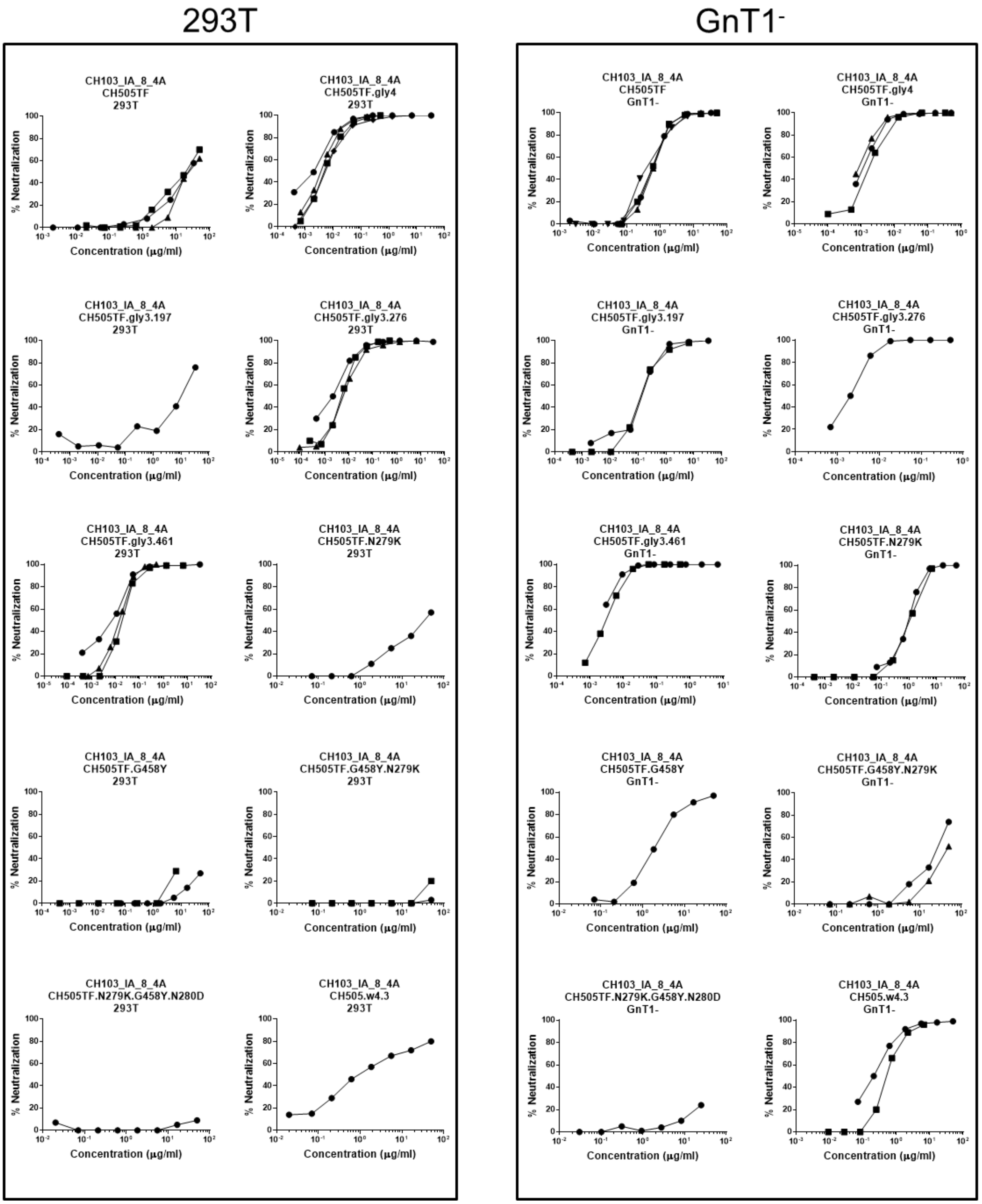

S1D Fig. Neutralization by CH103_IA_7_4A.

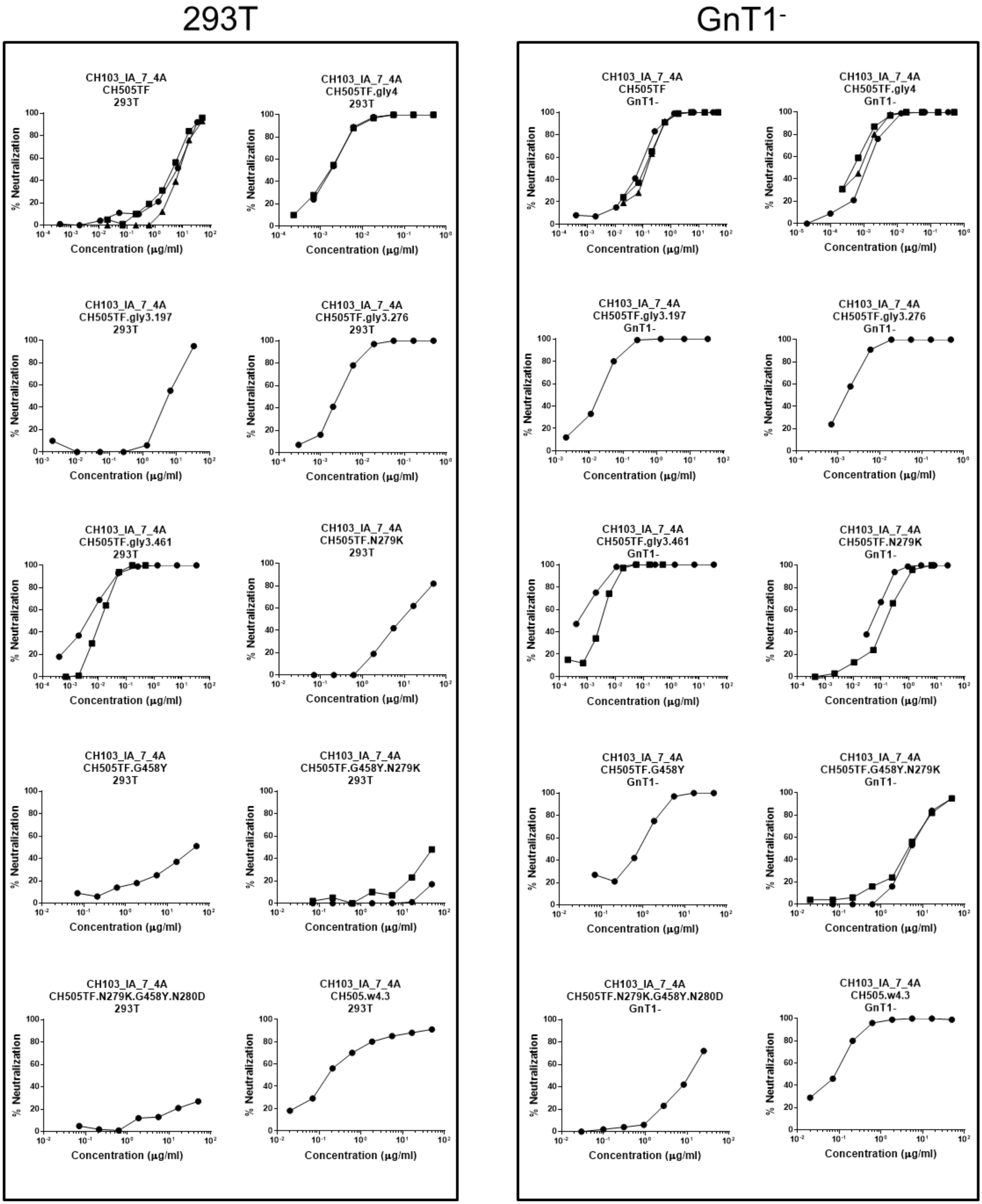

S1E Fig. Neutralization by CH103_IA_6_4A.

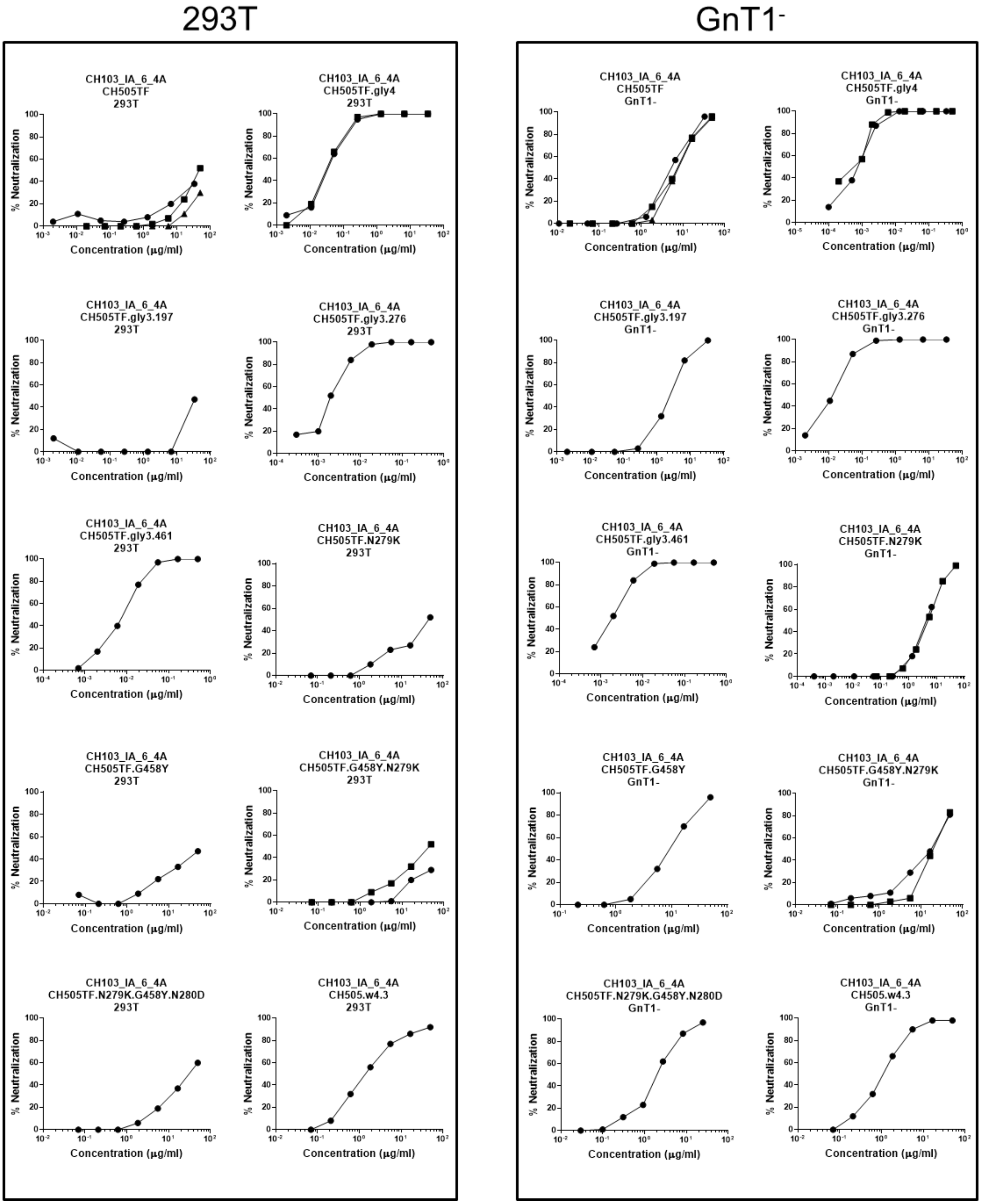

S1F Fig. Neutralization by CH103_IA_5_4A.

293T

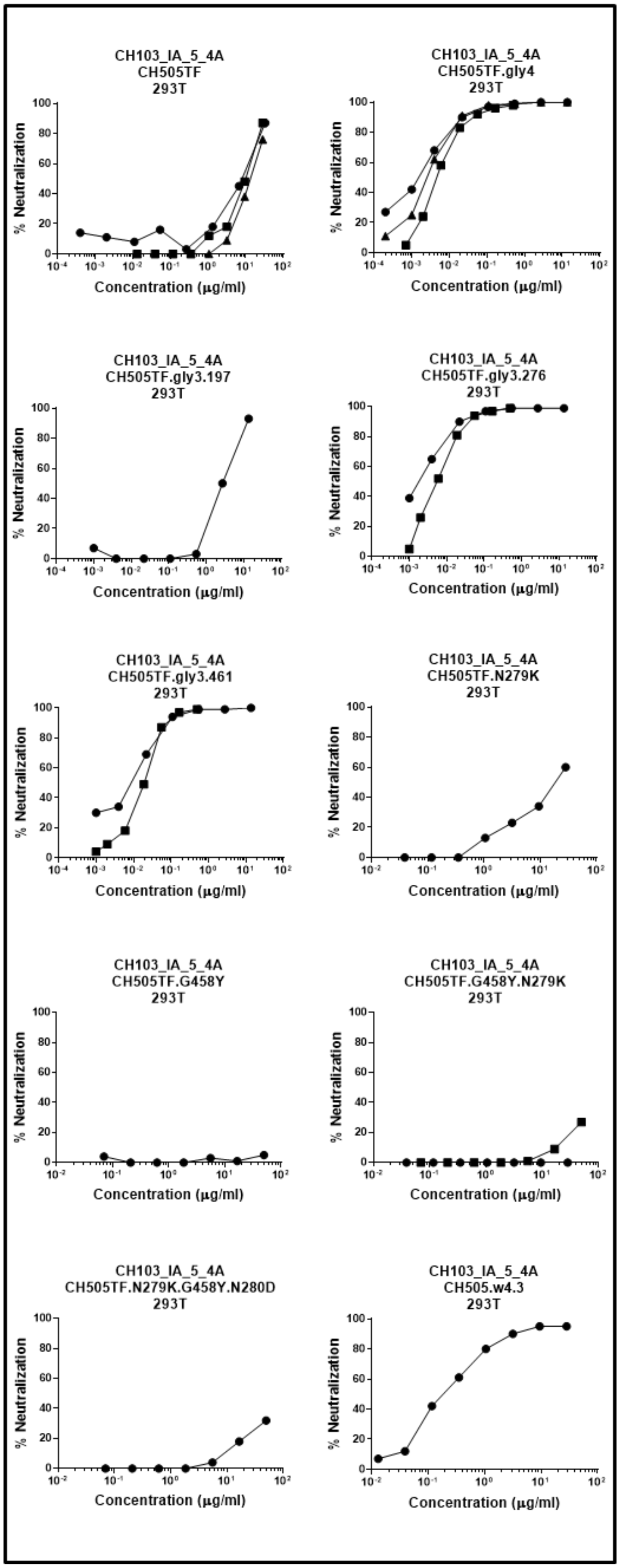

GnT1-

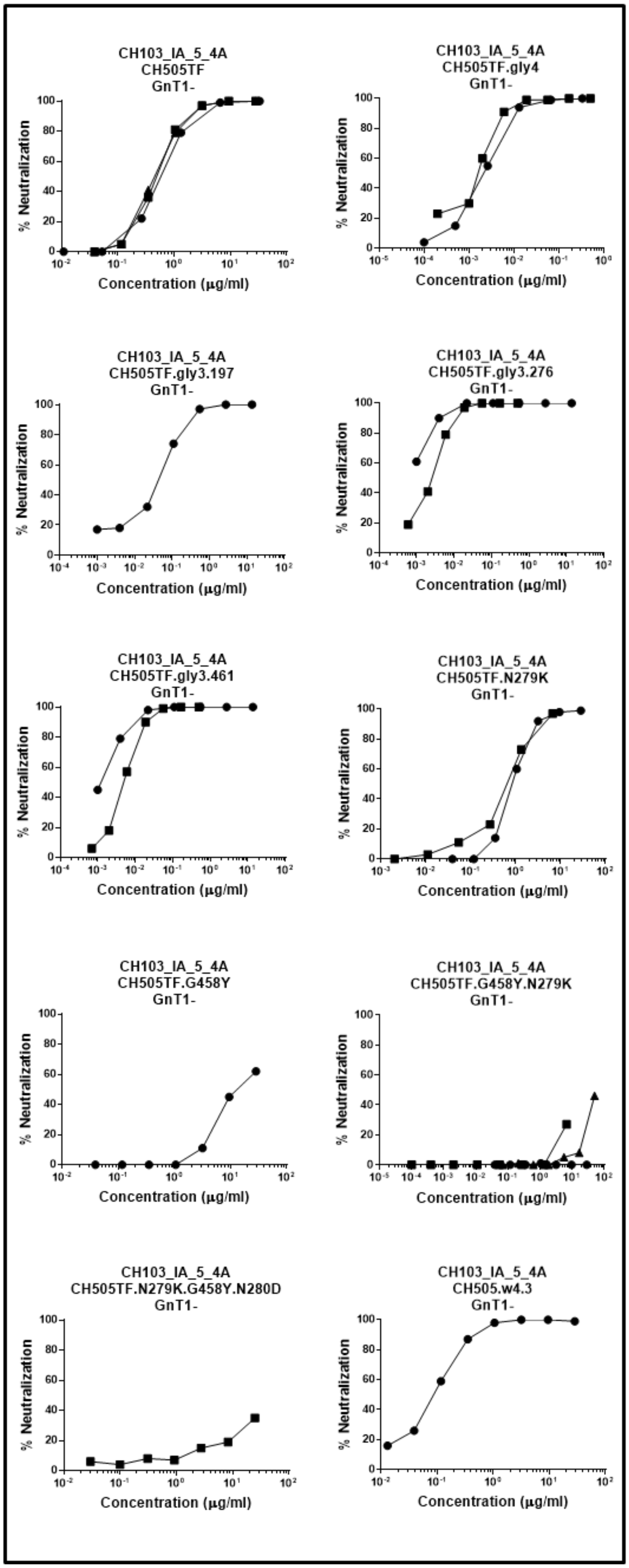

S1G Fig. Neutralization by CH103_IA_4_4A.

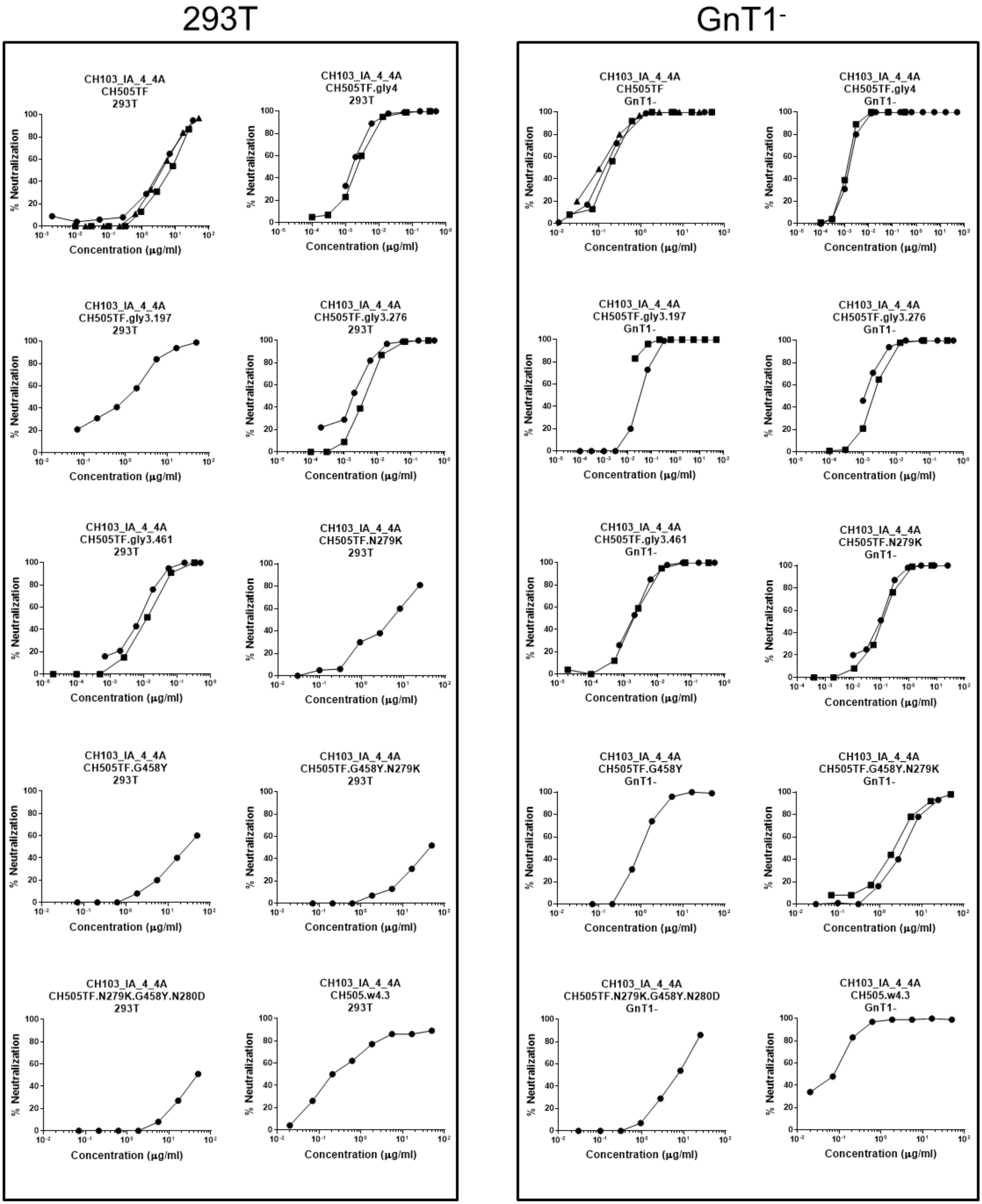

S1H Fig. Neutralization by CH235 UCA2

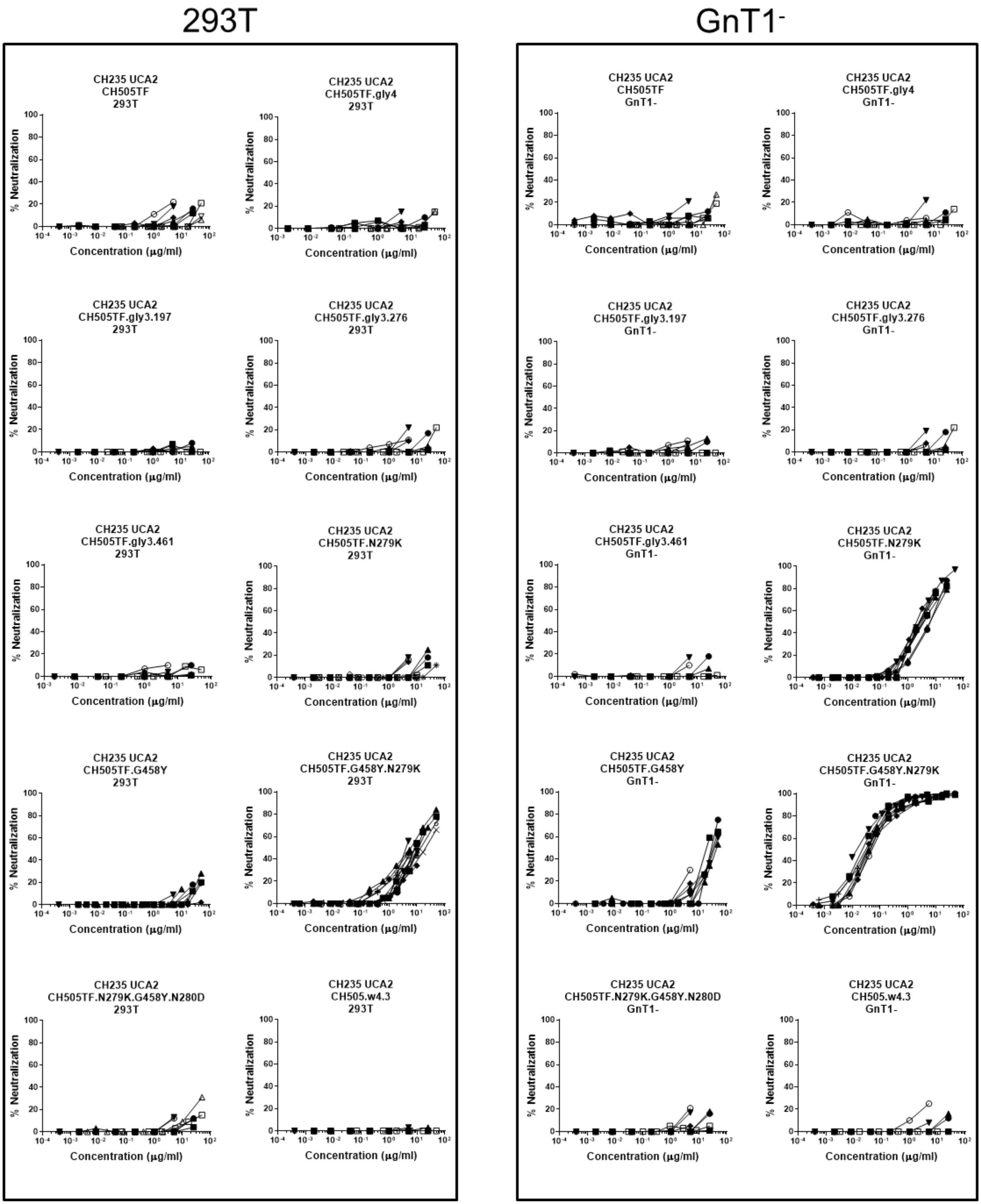

S1I Fig. Neutralization by CH235_I4_v2_4A

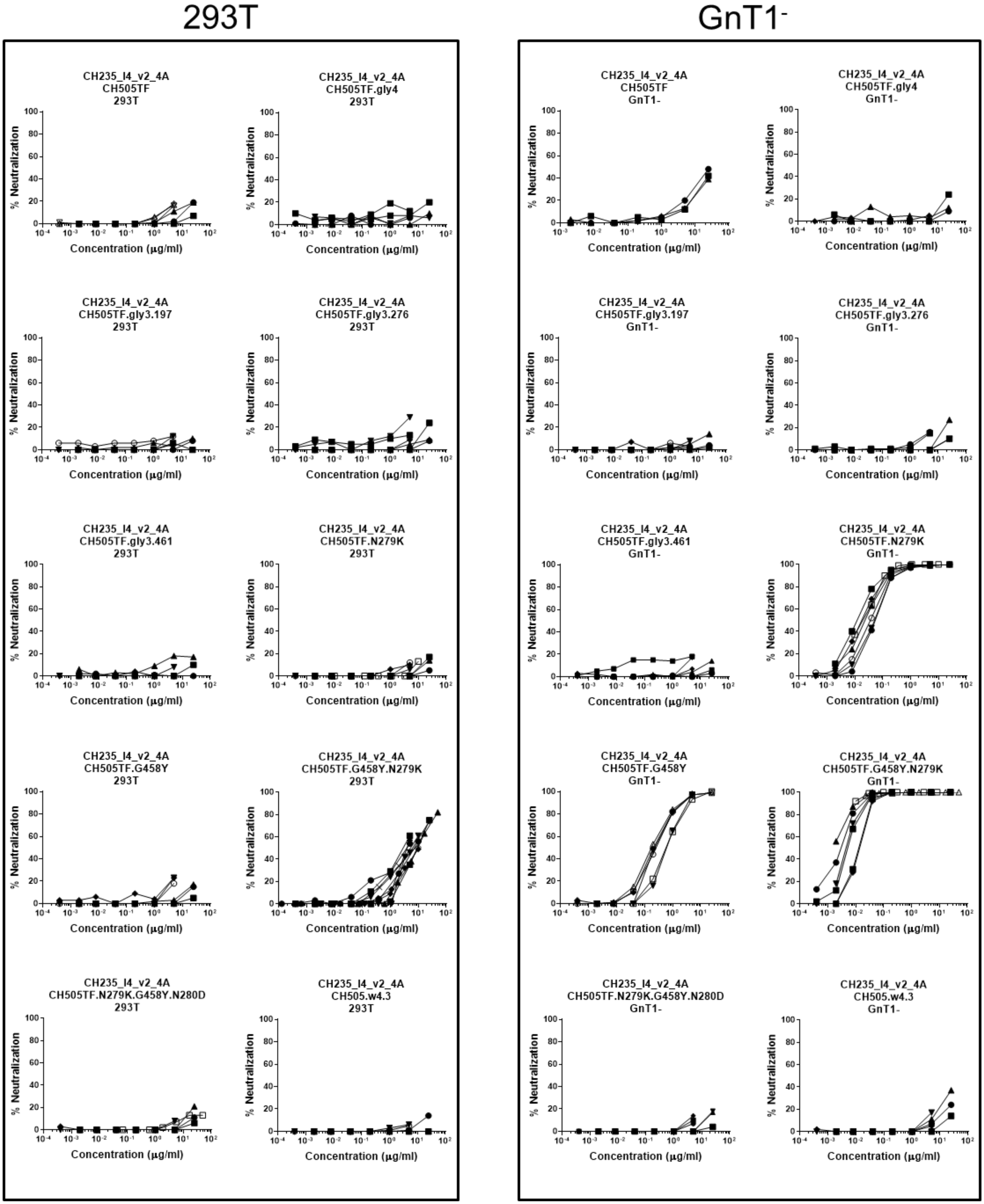

S1J Fig. Neutralization by CH235_I3_v2_4A

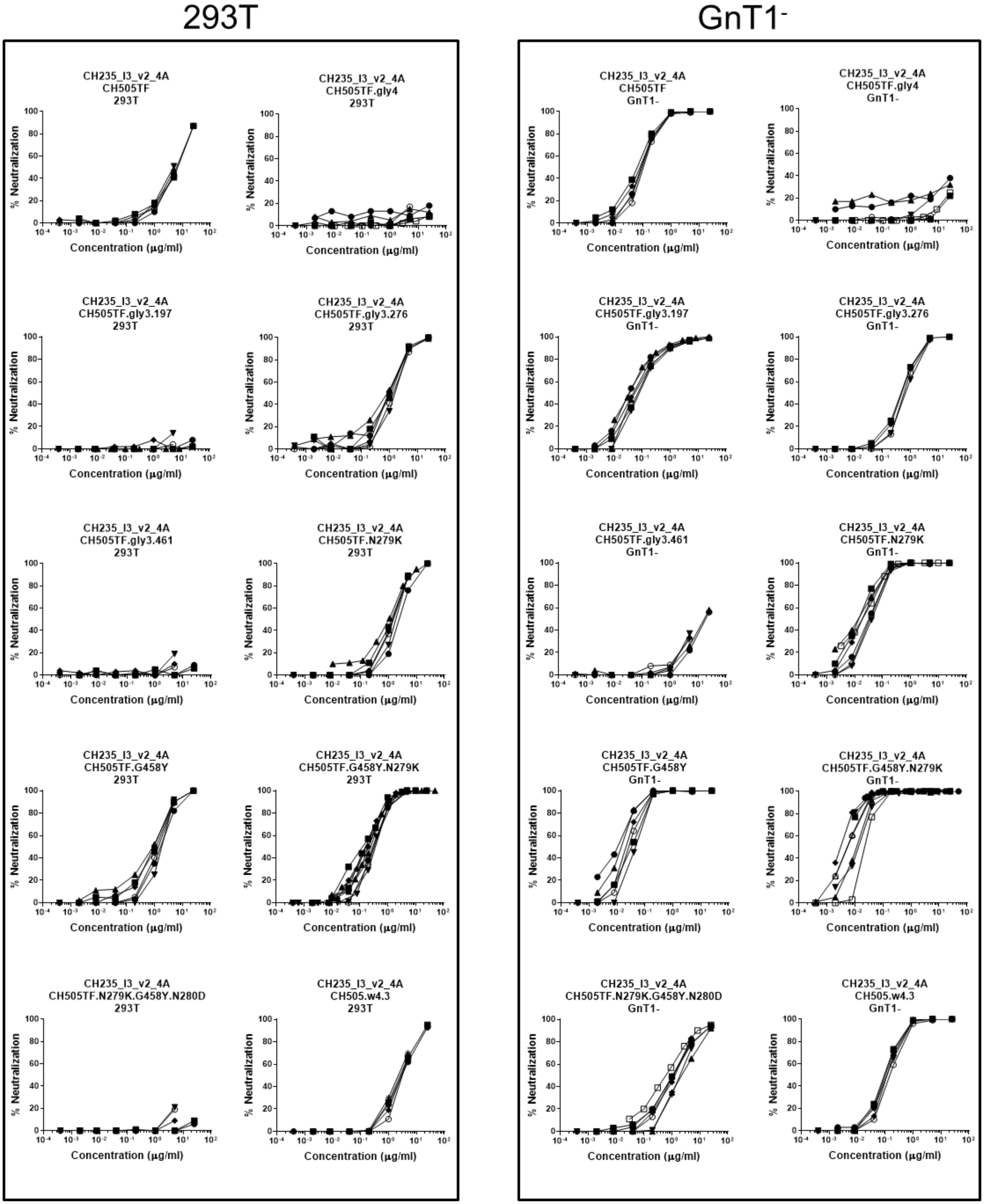

S1K Fig. Neutralization by CH235_I1_v2_4A

293T

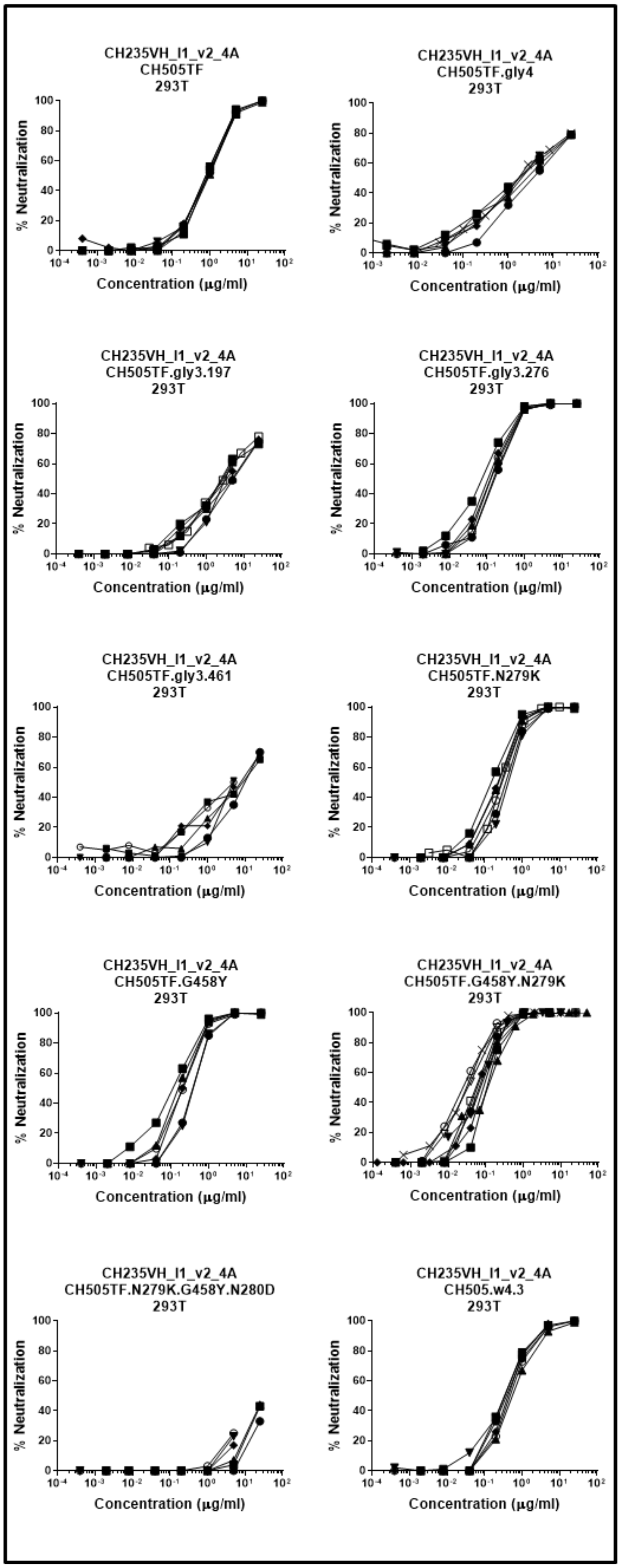

GnT1⁻

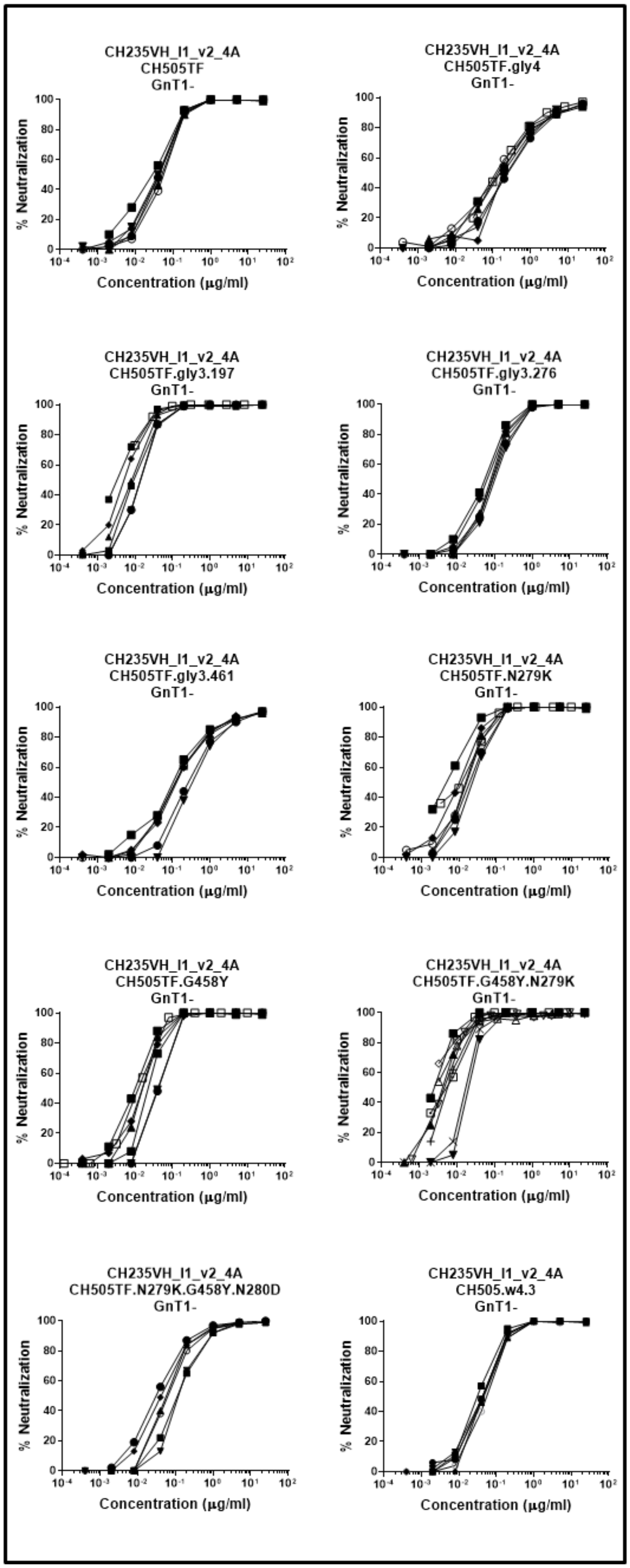

Supplement: S1 Fig — Neutralization assays were performed with Env-pseudoyped viruses prepared in either 293T or 293S/GnT1- cells and assayed in TZM-bl cells as described in Methods. The dilution factor (i.e., 3-fold or 5-fold) and range of bnAb concentrations evaluated varied depending on the potency of virus/antibody combinations, and sometimes multiple dilution factors and ranges were evaluated in repeat assays. This was done to obtain curves that were linear when crossing 50% neutralization for accurate measurement of IC50 values. When multiple assays were performed, the average IC50 was used in Fig 1. CH103_IA_9_4A is the earliest intermediate of this lineage tested while CH103_AI_4_4A is the latest. Likewise, CH235_I4_v2_4A is the earliest intermediate while CH235_I1_v2_4A is the latest intermediate tested. (A) CH103_UCA_4A; (B) CH103_IA_9_4A; (C) CH103_IA_8_4A; (D) CH103_IA_7_4A; (E) CH103_IA_6_4A; (F) CH103_IA_5_4A; (G) CH103_IA_4_4A; (H) CH235 UCA2; (I) CH235_I4_v2_4A; (J) CH235_I3_v2_4A; (K) CH235_I1_v2_4A. (PDF) [file ppat.1008026.s005.pdf]
