# Supplementary figures and images for: Neutralization-guided design of HIV-1 envelope trimers with high affinity for the unmutated common ancestor of CH235 lineage CD4bs broadly neutralizing antibodies
Source: PLoS Pathog. 2019 Sep 17;15(9):e1008026. doi: 10.1371/journal.ppat.1008026 (PMC6764681; doi:10.1371/journal.ppat.1008026)

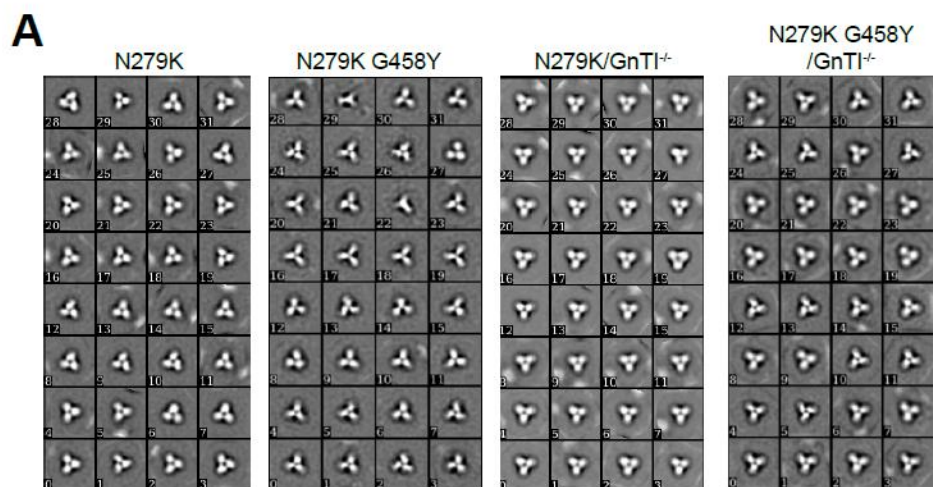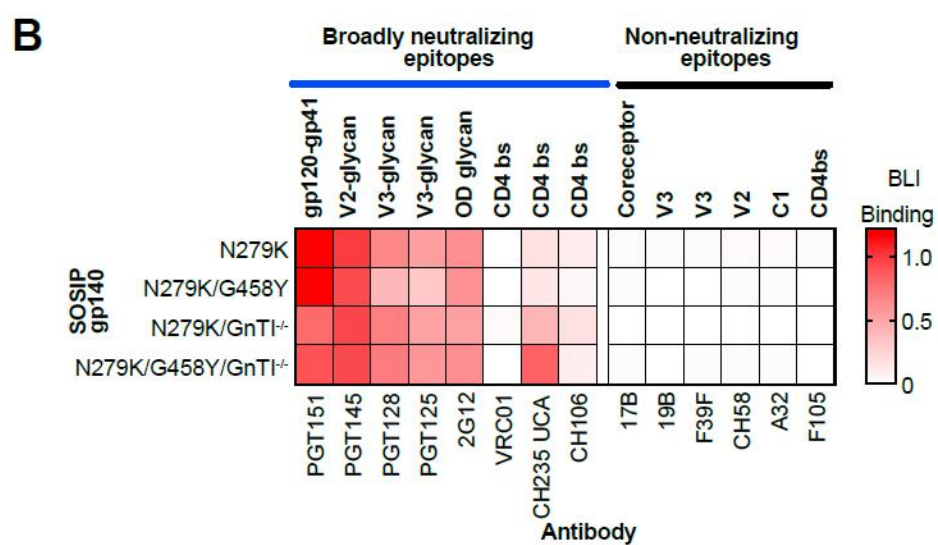

Supplement: S2 Fig — (A) Representative two-dimensional class averages of negative stain electron microscopy images of CH505 SOSIP gp140 proteins. The amino acid changes introduced into the CH505 transmitted/founder envelope sequence are indicated above each picture. (B) Antigenicity of each CH505 SOSIP gp140 variant as determined by biolayer interferometry (BLI). Values are binding responses in nm. GnT1- indicates proteins produced in 293S GnT1- cells to enrich for Man5GlcNac2. (PDF) [file ppat.1008026.s006.pdf]

# A CH505.N279K.G458Y.SOSIP.664/GnT1<sup>-</sup> + CH235 UCA2- Local Resolution

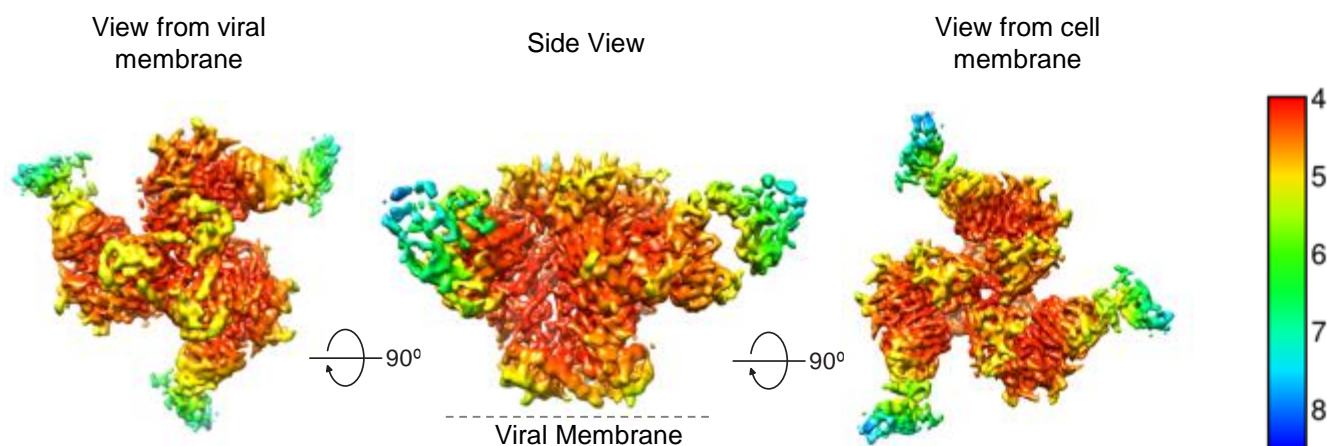

B

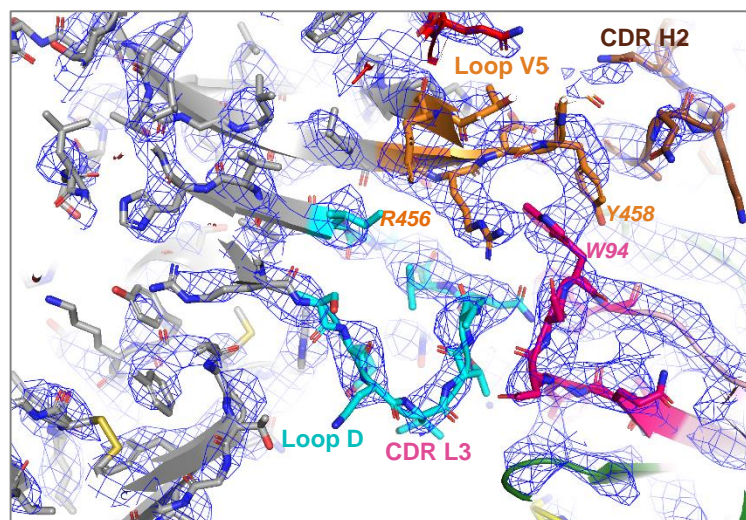

C

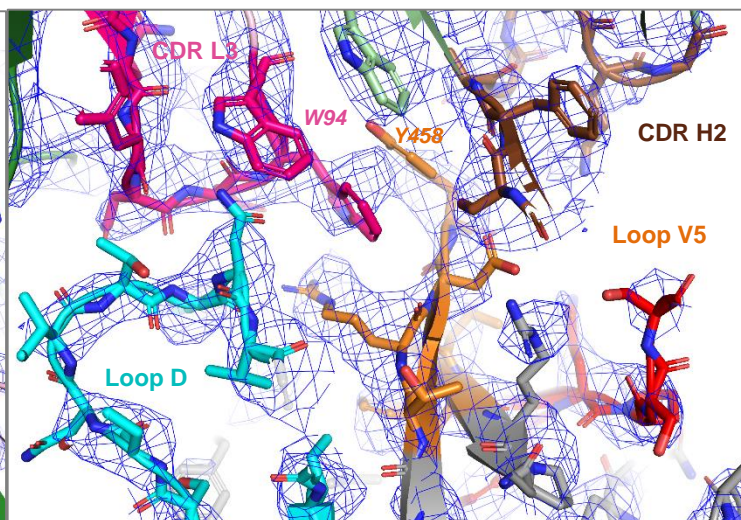

D

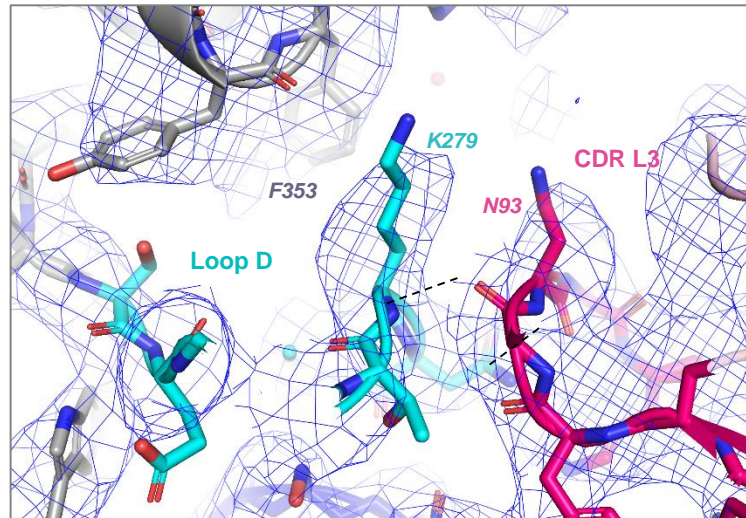

E

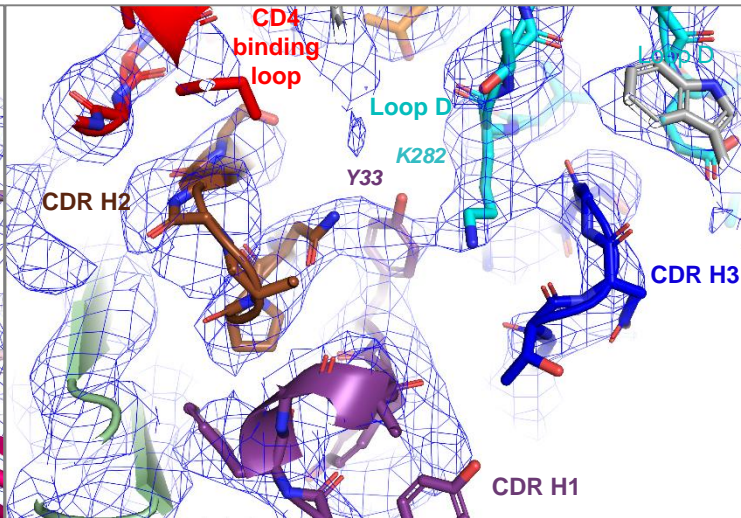

Supplement: S5 Fig — (A) Local Resolution color coded and plotted on the map surface (B-E) Zoomed-in views of the Loop V5 and Loop D regions of HIV-1 Env gp120 and the bound CH235 UCA2 antibody. The blue mesh indicates experimental cryo-EM density, and the underlying fitted model is shown in cartoon and stick representation. (PDF) [file ppat.1008026.s009.pdf]

**A**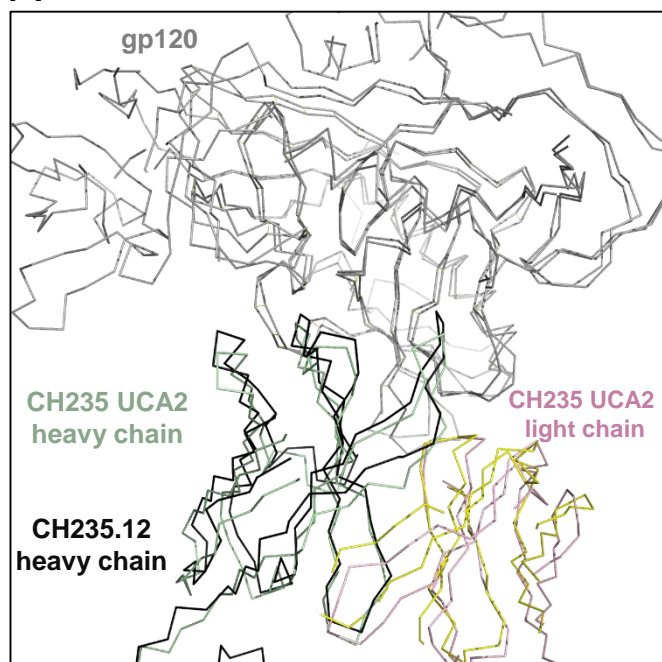**B**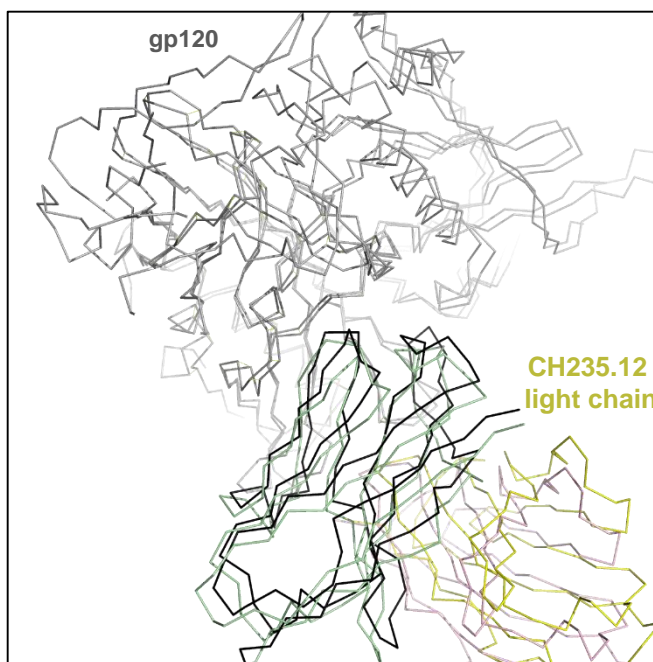**C**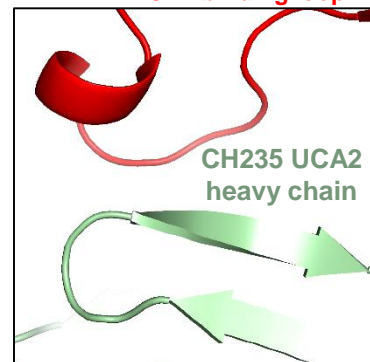**D**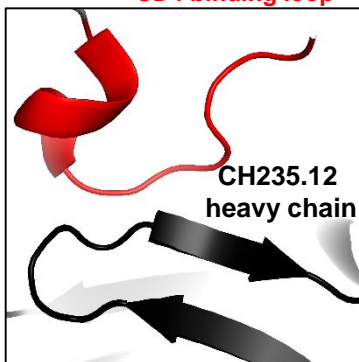**E**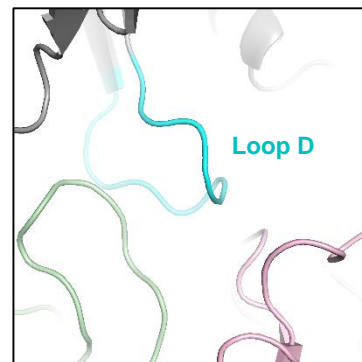**F**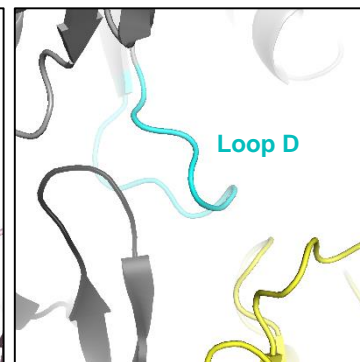**G**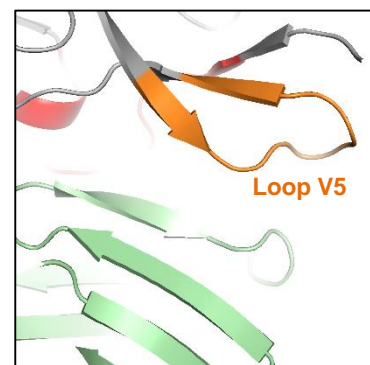**H**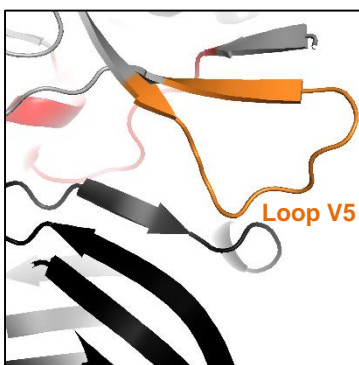**I**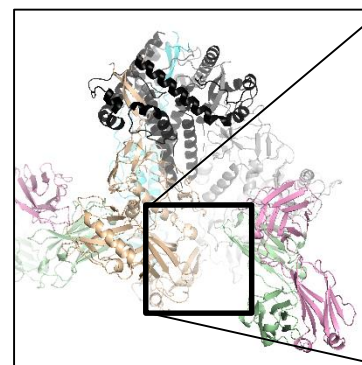**J**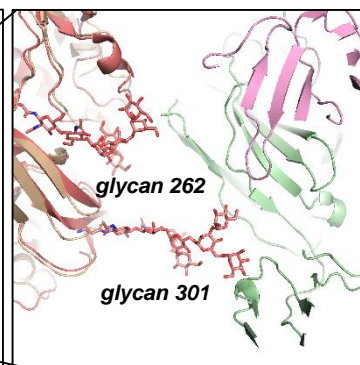

Supplement: S6 Fig — (A and B) Overlay of gp120 outer domains (gray) to show angles of approach of CH235 UCA2 and CH235.12 antibodies. (C-H) Comparison of CH235 UCA2 and CH235.12 binding to critical elements on gp120, with the CD4 binding loop shown in C and D, loop D shown in E and F, and loop V5 shown in G and H. (I) Env-bound CH235 UCA2 structure. (J) Zoomed-in view showing an overlay of gp120 from PDB ID 5FYL on the quaternary protomer. The SOSIP trimer in the 5FYL crystal structure was produced in 293F cells. Glycans 301 and 262 from the quaternary protomer, when in complex form, show close approach to CH235 UCA2. (PDF) [file ppat.1008026.s010.pdf]
