# Supplementary material for: Neutralization-guided design of HIV-1 envelope trimers with high affinity for the unmutated common ancestor of CH235 lineage CD4bs broadly neutralizing antibodies
Source: PLoS Pathog. 2019 Sep 17;15(9):e1008026. doi: 10.1371/journal.ppat.1008026 (PMC6764681; doi:10.1371/journal.ppat.1008026)
Supplement: S3 Fig — SPR affinity data are apparent KD values with rate constants derived from curve fitting analyses to 1:1 Langmuir model for binding of trimeric SOSIP proteins as analyte to CH235 UCA2 mAb immobilized on anti-Ig Fc mAb on the sensor chip. Concentration of SOSIP proteins in each binding curve is given. Data in table shown are results of two independent measurements by SPR analyses. (PDF) [file ppat.1008026.s007.pdf]

CH505TF SOSIP.664 v4.1_G458Y/GnT1^{-}

Assay 1 Assay 2

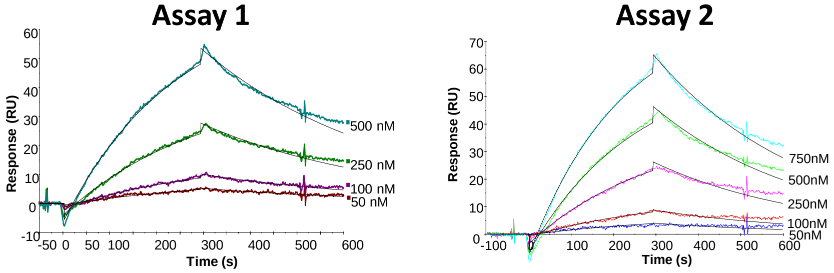

G458Y/GnT1-			
	K_a ($M^{-1}s^{-1}$)	k_d (s^{-1})	K_D (nM)
Assay 1	0.32×10^{4}	26×10^{-4}	822
Assay 2	0.24×10^{4}	28×10^{-4}	1170
Average			996

CH505TF SOSIP.664 v4.1_N279K/293F

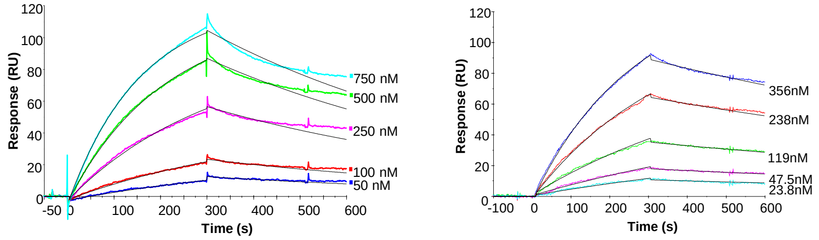

N279K/293F			
	K_a ($M^{-1}s^{-1}$)	k_d (s^{-1})	K_D (nM)
Assay 1	0.66×10^{4}	15×10^{-4}	231
Assay 2	0.91×10^{4}	7×10^{-4}	77
Average			154

CH505TF SOSIP.664 v4.1_N279K.G458Y/293F

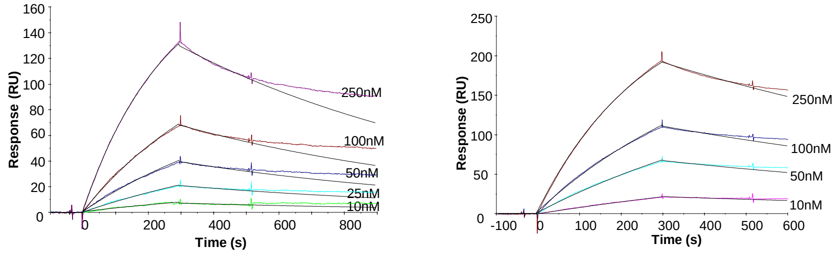

N279K.G458Y/293F			
	K_a ($M^{-1}s^{-1}$)	k_d (s^{-1})	K_D (nM)
Assay 1	1.17×10^{4}	10×10^{-4}	89
Assay 2	1.20×10^{4}	9×10^{-4}	77
Average			83

CH505TF SOSIP.664 v4.1_N279K/GnTI^{-}

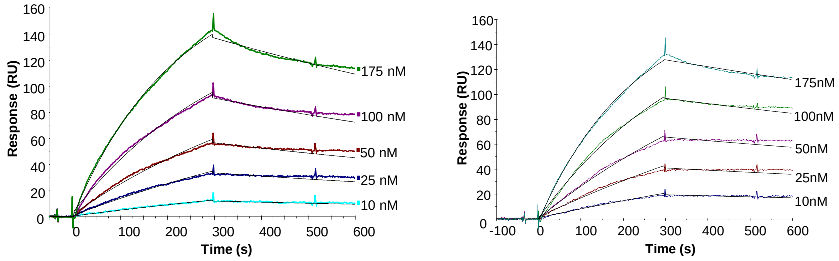

N279K/GnT1-			
	K_a ($M^{-1}s^{-1}$)	k_d (s^{-1})	K_D (nM)
Assay 1	1.18×10^{4}	8×10^{-4}	35
Assay 2	2.25×10^{4}	4×10^{-4}	20
Average			28

CH505TF SOSIP.664 v4.1_N279K.G458Y/GnTI^{-}

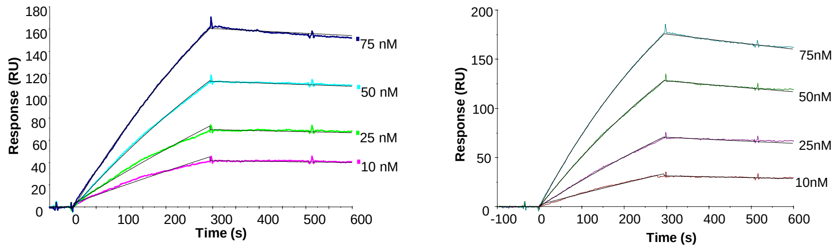

N279K.G458Y/GnT1-			
	K_a ($M^{-1}s^{-1}$)	k_d (s^{-1})	K_D (nM)
Assay 1	2.29×10^{4}	1×10^{-4}	6
Assay 2	2.70×10^{4}	3×10^{-4}	11
Average			9
