# Supplementary material for: Neutralization-guided design of HIV-1 envelope trimers with high affinity for the unmutated common ancestor of CH235 lineage CD4bs broadly neutralizing antibodies
Source: PLoS Pathog. 2019 Sep 17;15(9):e1008026. doi: 10.1371/journal.ppat.1008026 (PMC6764681; doi:10.1371/journal.ppat.1008026)
Supplement: S4 Fig — (A) Representative micrograph. (B) Initial 2D class averages showing larger complexes corresponding to Fab bound to Env trimer marked with red dots, and smaller complexes corresponding to a single Fab bound to an Env fragment, presumably gp120, marked with blue dots. (C) Final 2D class averages. (D) Ab initio model. (E) Refined map starting from ab initio generated model and refining it against a stack of cleaned-up particles, and applying C3 symmetry. (F) Fourier shell correlation curve. The dotted line indicates FSC0.143. (PDF) [file ppat.1008026.s008.pdf]

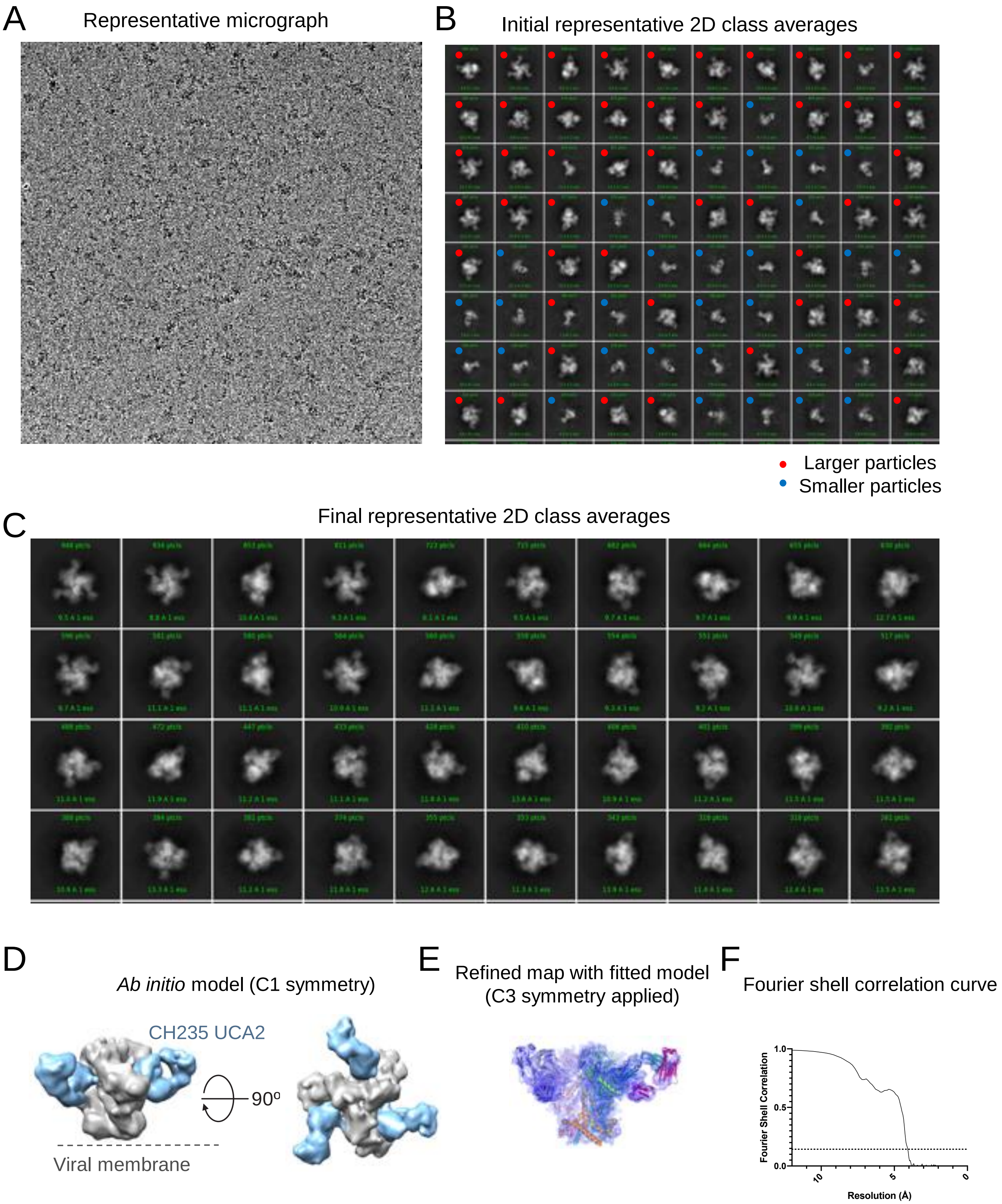

A
Representative micrograph
B
Initial representative 2D class averages
Larger particles
Smaller particles
C
Final representative 2D class averages
D
Ab initio model (C1 symmetry)
CH235 UCA2
90°
Viral membrane
E
Refined map with fitted model
(C3 symmetry applied)
F
Fourier shell correlation curve
Fourier Shell Correlation
1.0
0.5
0.0
10
5
0
Resolution (Å)
